# Single-molecule profiling of per- and polyfluoroalkyl substances by cyclodextrin mediated host-guest interactions within a biological nanopore

**DOI:** 10.1126/sciadv.adp8134

**Published:** 2024-11-06

**Authors:** Xiaojun Wei, Aditya Choudhary, Leon Y. Wang, Lixing Yang, Mark J. Uline, Mario Tagliazucchi, Qian Wang, Dmitry Bedrov, Chang Liu

**Affiliations:** ^1^ Department of Biomedical Engineering, University of South Carolina, Columbia, SC 29208, USA.; ^2^ Department of Chemical Engineering, University of South Carolina, Columbia, SC 29208, USA.; ^3^ Department of Materials Science and Engineering, University of Utah, Salt Lake City, UT 84112, USA.; ^4^ Department of Chemistry and Biochemistry, University of South Carolina, Columbia, SC 29208, USA.; ^5^ Department of Electrical and Electronic Engineering, The University of Melbourne, Parkville, VIC 3010, Australia.; ^6^ Facultad de Ciencias Exactas y Naturales, Departamento de Química Inorgánica Analítica y Química Física, Universidad de Buenos Aires, C1428 Ciudad Autónoma de Buenos Aires, Argentina.; ^7^ CONICET-Universidad de Buenos Aires, Facultad de Ciencias Exactas y Naturales, Instituto de Quimica de los Materiales, Ambiente y Energia (INQUIMAE), C1428 Ciudad Autonoma de Buenos Aires, Argentina.

## Abstract

Biological nanopores are increasingly used in molecular sensing due to their single-molecule sensitivity. The detection of per- and polyfluoroalkyl substances (PFAS) like perfluorooctanoic acid and perfluorooctane sulfonic acid is critical due to their environmental prevalence and toxicity. Here, we investigate selective interactions between PFAS and four cyclodextrin (CD) variants (α-, β-, γ-, and 2-hydroxypropyl-γ-CD) within an α-hemolysin nanopore. We demonstrate that PFAS molecules can be electrochemically sensed by interacting with a γ-CD in a nanopore. Using HP-γ-CDs with increased steric resistance, we can identify homologs of the perfluoroalkyl carboxylic acid and the perfluoroalkyl sulfonic acid families and detect common PFAS in drinking water at 0.4 to 2 parts per million levels, which are further lowered to 400 parts per trillion by sample preconcentration. Molecular dynamics simulations reveal the underlying chemical mechanism of PFAS-CD interactions. These insights pave the way toward nanopore-based in situ detection with promises in environmental protection against PFAS pollution.

## Teaser

A biological nanopore with cyclodextrin can detect polyfluoroalkyl substances (PFAS) in water.

## INTRODUCTION

Per- and polyfluoroalkyl substances (PFAS), such as perfluorooctanoic acid (PFOA) and perfluorooctane sulfonic acid (PFOS), are increasingly recognized as persistent and widespread environmental pollutants (*1*, *2*). These compounds, found in ground and surface waters, pose substantial health risks to many communities (*3*, *4*). In 2016, the United States Environmental Protection Agency (EPA) set a limit of 70 parts per trillion (ppt) for PFOA and PFOS in drinking water (*5*), but recent findings suggest that even lower concentrations can be harmful (*6*, *7*). In 2023, the EPA proposed the updated maximum contaminant levels of 4 ppt for both PFOA and PFOS, reflecting the latest scientific understanding of their long-term health impacts. The issued health advisories also highlight the growing concern over a broader range of PFAS molecules, such as perfluorobutanesulfonic acid (PFBS) and GenX chemicals (*8*), underscoring the need for more sensitive detection method effective remediation strategies. With the 2024 enforcement of these standards, the urgency of developing improved detection technologies has become more pronounced, emphasizing a crucial step toward mitigating this pervasive environmental health issue.

Now, the most reliable method for PFAS detection involves chromatography combined with mass spectrometry (*9*–*11*). Despite their sensitivity and specificity, these methods are strictly confined to laboratory settings due to their need for expensive equipment, specialized operators, complex sample preparation, and extended analysis times (*12*). In addition, the stubborn persistence of PFAS residues can diminish the sensitivity of these instruments over time (*13*, *14*). As a result, there has been a substantial push for developing alternative detection methods that are simpler, more accessible, cost-effective, and rapid. Representative studies include a guanidinocalix[5]arene-assisted fluorescent indicator (*1*), a near-infrared excitation surface molecular imprinting ratiometric fluorescent probe (*15*), and MoS_2_/Fe_3_O_4_ nanocomposites for colorimetry (*16*), as well as deep eutectic solvent-based superparamagnetic nanofluid (*17*), metal-organic framework-based microfluidic impedance sensor (*18*), a bubble nucleation–based method (*19*), etc. While these methods primarily rely on optical signals like fluorescence and absorbance, they often require complex synthetic sensing probes and extensive efforts to enhance PFAS selectivity (*2*).

As detection methods evolve, additional regulations for treating and discharging organic pollutants are being established. Owing to the challenge of breaking the strong C─F bond (bond energy ≈ 110 kcal/mol) in fluorocarbons to transform PFAS into harmless substances, the current primary treatment strategy is adsorption by various adsorbents (*20*). Cyclodextrins (CDs), cyclic oligosaccharides with a hydrophilic exterior and a hydrophobic cavity, have emerged as effective adsorbents in environmental applications (*21*). They solubilize and remove organic pollutants from soil and water (*22*). For example, β-CD polymer networks (*3*) and iron-montmorillonite-CD composites showed promising results in treating PFOA (*21*).

Meanwhile, nanopore technologies, known for their high sensitivity and nondestructive nature, are gaining prominence in various fields, including environmental monitoring (*23*–*27*). Over the past two decades, nanopore has rapidly advanced as a highly sensitive single molecule sensing technology, surpassing traditional fluorescence methods (*28*). Some nanopore studies have integrated CDs, particularly β-CD and γ-CD, as molecular adaptors for various analytes due to their unique cavity structures (*29*–*32*). Accordingly, CDs can noncovalently fit into the nanopore stem, serving as an adaptor for sensing DNA nucleotides (*33*), nanoparticles (*34*), polysulfides (*35*), ester hydrolysis (*36*), and other organic molecules (*29*). However, the use of mutant protein nanopores and modified CDs in these pioneering works presents challenges for non-expert nanopore users, thus prevent field applications (*37*, *38*). In a nanopore sensor system, a thin lipid membrane containing a protein nanopore divides an ionic solution into two compartments. Analytes are transported across the membrane through the nanopore under a transmembrane voltage while being measured (*39*, *40*). Inspired by the selective adsorption of PFAS to CDs and their role in nanopore-based molecular sensing, we hypothesize that the host-guest interactions between CDs and PFAS molecules can disrupt the current in the nanopore, allowing us to detect PFAS molecules by analyzing the altered signals.

Here, by using an α-hemolysin (α-HL) nanopore, the host-guest interactions between molecules of the PFOA and PFOS families and four different CDs: α-CD, β-CD, γ-CD, and 2-hydroxypropyl-γ-CD (HP-γ-CD), are systematically investigated. Our approach enables single-molecule detection of PFAS molecules through their interaction with γ-CD within an α-HL nanopore. Notably, using HP-γ-CDs with enhanced steric resistance, we identified homologs of the perfluoroalkyl carboxylic acid (PFCA) and perfluoroalkyl sulfonic acid (PFSA) families. Furthermore, we demonstrate the detection limits of common PFAS molecules in drinking water at parts per million (ppm) levels by direct detection and at ppt levels following sample preconcentration. Through experimental investigation, ion transport modeling, and molecular dynamics (MD) simulations, we attempt to unravel the sensing mechanism. This strategy shows promise for developing a portable device for in situ PFAS pollution monitoring.

## RESULTS

### Engineering CD-mediated nanopore sensing for PFAS

**Fig. 1.**
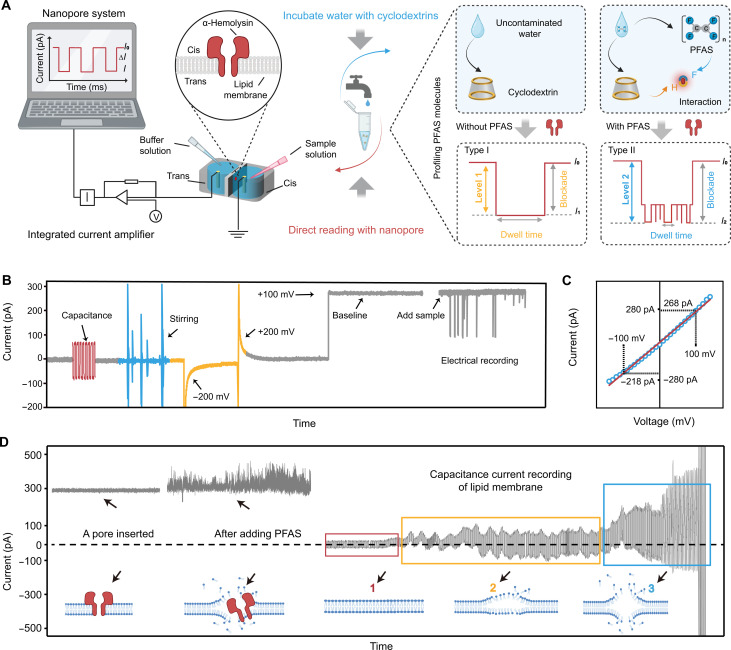
Engineering an α-HL nanopore system with CD for sensing PFAS molecules. (**A**) Schematic illustration of the experimental setup (left) and the CD mediated nanopore assay workflow for profiling PFAS molecules (right). Left: A nanopore system. An α-HL protein nanopore is inserted into a lipid bilayer membrane suspended across the 200-μm aperture on a Delrin wall that divides a chamber into two compartments: cis and trans. A command voltage is applied on the trans side, and the cis side is grounded. An integrated current amplifier is used for measuring transient current blockages caused by translocation of sample molecules after adding the sample into the cis side. Right: PFAS molecule profiling. The samples are achieved by incubating water possibly containing PFAS with CD followed by nanopore measurement of current blockage events for PFAS profiling and quantification. (**B**) Typical nanopore current traces of a stable lipid bilayer membrane by using capacitance monitor, stirring in cis side, and flipping the applied cross-membrane voltage between ±200 mV, together with the typical baseline after a nanopore is inserted into the membrane and the typical current trace after adding analytes into the cis side of chamber. (**C**) Single-channel current-voltage curve of an open α-HL nanopore in 3 M KCl. (**D**) The raw current trace of a blank buffer after an α-HL nanopore inserted into the lipid membrane and the typical fluctuant current trace after adding PFAS molecules into the cis side of chamber. The capacitance current trace of a lipid bilayer membrane records the process of membrane transitioning from an unstable state to rupture under the attack of PFAS molecules. 1: A stable membrane with a thickness of 4.9 nm; 2: Membrane is affected by PFAS molecule interference; 3: Membrane gradually thins until it ruptures. Insets are corresponding schematic diagrams (not to scale).

Figure 1A illustrates the principle of electrochemical nanopore sensing for single-molecule PFAS detection. Measurements were conducted using a single biological nanopore setup, comprising two compartments (cis and trans) separated by a self-assembled α-HL in a lipid bilayer membrane, immersed in a KCl electrolyte (*41*). Ag/AgCl electrodes in both chambers, connected to a current amplifier, allowed for the application of a transmembrane potential with the cis chamber grounded and the measurement of the ionic current going through the nanopore (*42*). Upon introducing a sample into the cis chamber, a time-varying current is recorded. For stochastic sensing of PFAS, CDs (i.e., γ-CD or HP-γ-CD) are first mixed with potentially contaminated water to form CD-PFAS complexes. These complexes are then introduced into the cis chamber, where CD-PFAS host-guest interactions manifest as resistive pulse signals in the ionic current. Applying a constant transmembrane potential bias in the absence of any analytes enables a stable picoampere ionic current flow through the unblocked α-HL (*I*_0_, the open pore current) as a baseline. As CD molecules either pass through or block the nanopore, each molecule induces a noticeable decrease in this baseline current (level 1 ∆*I* = *I*_0_ − *I*_1_) (Fig. 1A, type I). In addition, when PFAS molecules present in the system, a further reduction in current occurs (level 2 *∆I* = *I*_0_ − *I*_2_), appearing as a mutilevel current blockade (Fig. 1A, type II). These distinct, highly reproducible blockades (level 1 *∆I* and level 2 *∆I*), resulting from the host-guest interaction between the PFAS and the CD molecules, act as unique signatures to enable the detection of various PFAS via the nanopore sensor.

Before each measurement, the capacitance of the lipid membrane was recorded to ensure proper formation. The membrane stability was verified by flipping the applied voltage (±200 mV) and magnetically stirring the electrolyte solution in the cis chamber (Fig. 1B and fig. S1). When a single α-HL nanopore self-assembles in the membrane with a +100-mV voltage, an instant current increase can be observed for confirmation (Fig. 1B, baseline). The current-voltage relationship further validates the nanopore’s orientation and configuration, with larger ionic currents observed at positive trans voltages than at negative trans voltages (i.e., 268 pA under +100 mV and −218 pA under −100 mV) (Fig. 1C) (*25*, *43*). After these quality control steps, a sample can be added to the cis side for signal acquisition.

Before investigating interactions between PFOA/PFOS and CDs, we first evaluated the effects of unbound PFOA/PFOS on the nanopore system. As strong acids, PFOA [weight-average molecular weight (*M*_w_): 414.07 g/mol] and PFOS (*M*_w_: 500.03 g/mol) fully dissociate into anions and hydrogen ions in water, remaining negatively charged at pH 8. Following the addition of PFOA or PFOS to the cis side (0.1 mM final concentration) under a +100-mV constant voltage, notable fluctuations in the baseline current occurred in a short period (Fig. 1D and figs. S2 and S3). The disruption was further quantified by monitoring lipid membrane’s capacitance. Initially stable at 171 ± 22.19 pF, correlating to a thickness of 4.9 ± 0.72 nm (*44*), the capacitance increased upon PFOA or PFOS introduction, signaling a decrease in membrane thickness and eventual rupture. Above observations confirm that PFOA and PFOS notably disrupt lipid membrane stability, aligned with their known surfactant properties that compromise membrane structures (*45*, *46*). To assess PFOA and PFOS interactions with the nanopore, we reduced their concentrations and noted that, at 0.001 mM, the stability of membrane was improved, enabling effective recording of distinct PFOA and PFOS signals (fig. S4). However, these signals were hard to differentiate from occasional noise signals and from each other, thus cannot support reliable identification and quantification. These findings suggest inherent challenges and limitations in directly detecting naked PFOA and PFOS molecules via nanopore sensing.

Inspired by prior research, we explored various CDs as “sensing adaptors” for PFAS in the nanopore. Given the diameter of CF_2_ (0.5 nm) and CF_3_ (0.7 nm) groups in PFAS, CDs with cavities of ≥0.7 nm can potentially encapsulate these groups (*47*, *48*). We assessed four CDs (i.e., α-CD, β-CD, γ-CD, and HP-γ-CD) of different sizes and structures (fig. S5) for their efficacy in detecting and differentiating PFAS, specifically PFOA and PFOS families. Our findings revealed that γ-CD and HP-γ-CD enabled the nanopore system to generate characteristic PFAS signals.

### Host-guest interactions between PFOA or PFOS and γ-CD within an α-HL nanopore

**Fig. 2.**
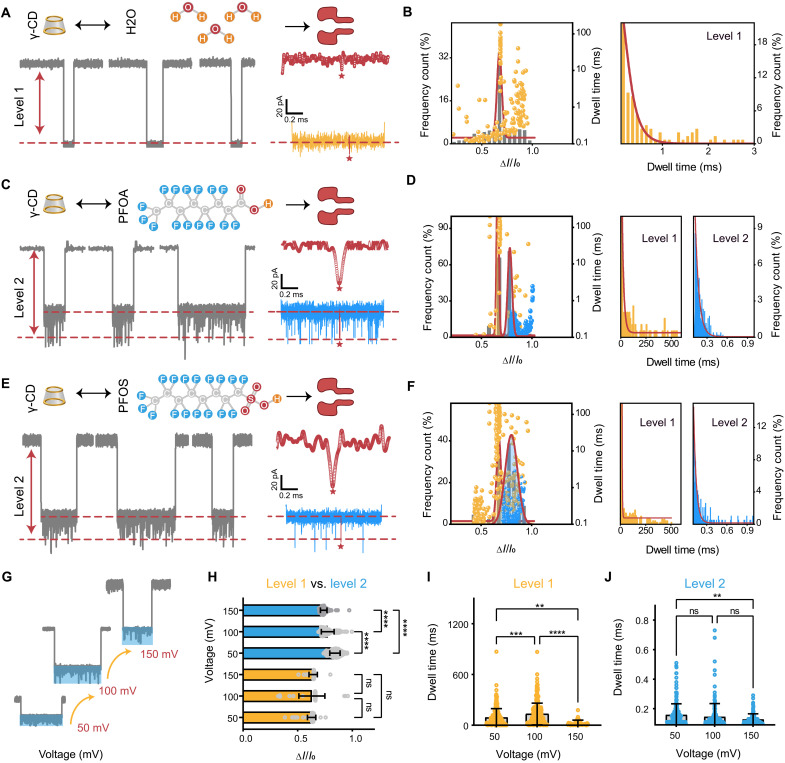
Electrochemical profiling of PFOA and PFOS by γ-CD–mediated host-guest interactions within an α-HL nanopore. (**A**, **C**, and **E**) Illustration of the interaction between (A) γ-CD and H_2_O, (C) γ-CD and PFOA, and (E) γ-CD and PFOS with an α-HL nanopore (top) and close-ups of representative current traces and corresponding magnified areas of blockage events (bottom). The red stars denote the typical blockages magnified. (**B**, **D**, and **F**) Profiling of the interaction between (B) γ-CD and H_2_O, (D) γ-CD and PFOA, and (F) γ-CD and PFOS within an α-HL nanopore (detailed information shown in table S1). Left: Corresponding two-dimensional scatterplots of relative blockade versus dwell time of valid level 1 (yellow) and level 2 (blue) events from nanopore results and histograms of normalized event frequency versus current blockade. The red solid curves show Gaussian fits. Right: Dwell time histograms of level 1 and level 2 events. The red solid curves are single exponential fits to the histograms. (**G** to **J**) Effects of the applied voltage on the interaction of γ-CD and PFOA. (G) Representative current traces produced by γ-CD-PFOA in an α-HL nanopore under +50-, +100-, and + 150-mV bias voltages. Bar graphs of corresponding current blockade (H) and dwell time (I and J) of level 1 and level 2 events (**0.001 < *P* < 0.01, ***0.0001 < *P* < 0.001, and ****P < 0.0001). ns, not significant.

At +100-mV trans potential, γ-CD was first introduced to the cis side of α-HL nanopore. The internal and the external diameters of γ-CD are 0.77 and 1.69 nm (*49*), allowing it to enter the ~3.6-nm-wide vestibule from the 2.4-nm opening of an α-HL but unable to pass through the narrowest region (~1.4-nm wide) of α-HL to enter the trans side (*50*, *51*). Thus, only a few water molecules could enter the hydrophobic vestibule of γ-CD during its entire interaction process with the nanopore (Fig. 2A, top). This resulted in regular ionic current blockages [Fig. 2A (bottom) and fig. S6] with a normalized blockade ratio of 0.674 ± 0.048 and a duration of 0.185 ± 0.002 ms (Fig. 2B). The neutrality of γ-CDs suggests that electroosmotic flow is the main driving force behind the insertion of this CD into the α-HL nanopore (*49*). Notably, the blockage characteristics transformed considerably after introducing PFOA and PFOS molecules (Fig. 2, C and E, and figs. S7 and S8), manifesting a secondary signal (level 2) while maintaining similar level 1 blockade ratios: 0.674 ± 0.048 for γ-CD, 0.661 ± 0.023 for γ-CD-PFOA, and 0.676 ± 0.043 for γ-CD-PFOS (Fig. 2, D and F). The presence of PFAS molecules subtly modified the interaction, evident from the unchanged level 1 blockade but extended dwell times compared to solo γ-CD: 0.185 ± 0.002 ms for γ-CD, 16.7 ± 2.001 ms for γ-CD-PFOA, and 2.63 ± 0.145 ms for γ-CD-PFOS. The increase in dwell time could stem from increased volume and electronegativity resulting from the binding of PFAS to the γ-CD molecule and the influence of free PFAS on the equilibrium of this reversible binding. Further analysis revealed distinct secondary signal characteristics: γ-CD-PFOA showed a blockade of 0.781 ± 0.042 and a dwell time of 0.055 ± 0.002 ms (Fig. 2D); γ-CD-PFOS showed a blockade of 0.799 ± 0.121 and a dwell time of 0.038 ± 0.001 ms (Fig. 2F). These differences suggest that PFAS molecules uniquely interact with γ-CD’s cavity, altering its blockage process within the α-HL pore and resulting in temporary reductions in current that manifest as distinctive level 2 signals. As observed in previous studies, similar signals also reflect binding and dissociation dynamic between analytes and CDs (*52*, *53*).

The blockade and dwell time behaviors of molecules in a nanopore are known to be voltage sensitive (*49*). By investigating γ-CD-PFOA within an α-HL nanopore (Fig. 2G and fig. S9), we note that with a voltage increment from +50 to +150 mV, the level 1 blockade (γ-CD alone) remained fairly stable between 0.615 ± 0.022 and 0.675 ± 0.017. Conversely, the level 2 blockade (indicative of PFOA sensing) decreased from 0.873 ± 0.076 to 0.751 ± 0.057 (Fig. 2H and fig. S10).

Both levels’ dwell times notably reduced at higher voltages (Fig. 2, I to J, and table S1), and the frequency of multilevel signals notably dropped (fig. S9), particularly at +150 mV. No substantial secondary signals were detected at voltages below +50 mV. These observations suggest that the host-guest interaction between CD and PFAS inside an α-HL nanopore is voltage dependent. Both very low and very high voltages are proven to be detrimental for effectively monitoring these interactions. Consequently, a standardized voltage of +100 mV was chosen for subsequent measurements.

### HP-γ-CD–mediated PFOA and PFOS profiling by enhanced host-guest interaction

**Fig. 3.**
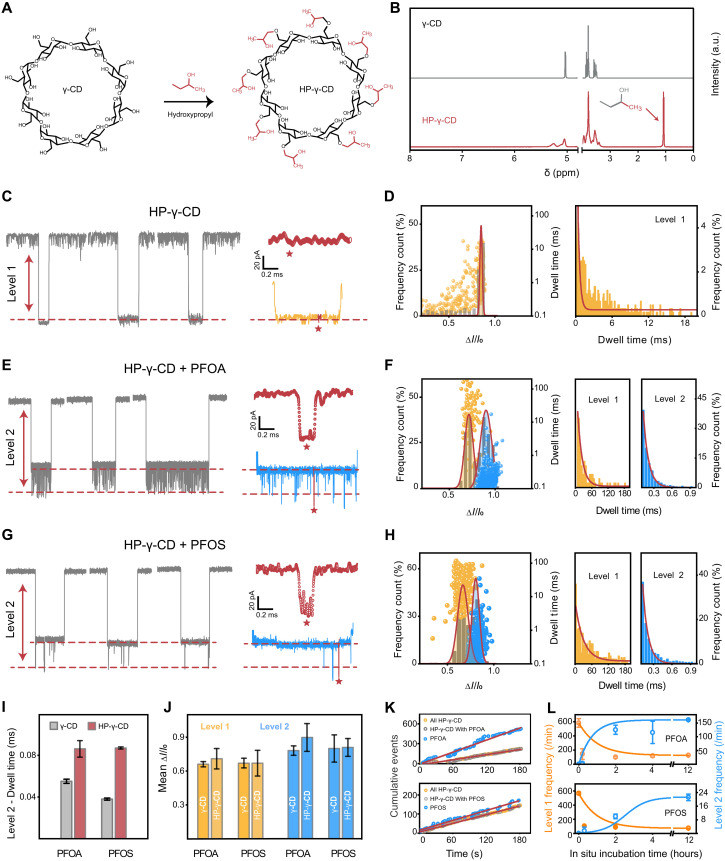
Enhanced electrochemical profiling of PFOA and PFOS by HP-γ-CD–mediated host-guest interactions within an α-HL nanopore. (**A** and **B**) Chemical structures (A) and corresponding ^1^H-NMR spectra (B) of γ-CD and HP-γ-CD. (**C**, **E**, and **G**) Close-ups of representative current traces and corresponding magnified areas of blockage events of interaction between (C) HP-γ-CD and H_2_O, (E) HP-γ-CD and PFOA, and (G) HP-γ-CD and PFOS within an α-HL nanopore. The red stars denote typical blockades. (**D**, **F**, and **H**) Profiling of the interaction between (D) HP-γ-CD and H_2_O, (F) HP-γ-CD and PFOA, and (H) HP-γ-CD and PFOS within an α-HL nanopore (detailed information shown in table S1). Left: Scatterplots of relative blockade versus dwell time of valid level 1 (yellow) and level 2 (blue) events from nanopore results and histograms of normalized event frequency versus current blockade. The red solid curves show Gaussian fits. Right: Dwell time histograms of events. The red solid curves are single exponential fits. (**I**) Comparison of the dwell time of level 2 signals induced by interactions between PFOA/PFOS and γ-CD/HP-γ-CD. (**J**) Comparison of the current blockade of level 1 and level 2 signals induced by interactions between PFOA/PFOS and γ-CD/HP-γ-CD. The error bars in (I) to (J) represent the SD of Δ*I*/*I*_0_ from the Gaussian fitting. (**K**) Cumulative events of different species in HP-γ-CD–PFOA and HP-γ-CD–PFOS systems acquired using nanopore over the recording period. The red solid curves show linear fits. (**L**) Kinetic studies: Time-dependent statistical frequencies and corresponding SDs (error bars) of level 1 and level 2 signals induced by interactions between PFOA/PFOS and HP-γ-CD in a nanopore, respectively. Note: Level 1 frequency represents the total number of individual CD molecular events per unit time, in which no secondary signals are observed. Level 2 frequency refers to the total number of secondary signals generated by PFOA or PFOS per unit time. a.u., arbitrary units.

Above results demonstrate that the interaction between γ-CD and PFOA or PFOS molecules facilitates characteristic nanopore signals of them, suggesting a potential PFAS sensor. Yet, distinguishing between PFOA and PFOS based on their secondary signals is difficult with γ-CDs. We hypothesized that enhancing host-guest interaction through γ-CD modifications, such as hydroxypropyl group, could provide specific structural cues for differentiating various types of PFAS. To this end, further experiments were conducted using HP-γ-CD instead of γ-CD. ^1^H–nuclear magnetic resonance (NMR) spectra (Fig. 3, A and B) confirmed the structural distinction between γ-CD and HP-γ-CD, with a unique peak near 1.1 ppm indicative of the hydroxypropyl group (*54*).

Compared to γ-CD, HP-γ-CD exhibits similar blockage events within an α-HL nanopore with a larger blockade value of 0.863 ± 0.037, reflecting the impact of additional hydroxypropyl groups on the ion transport (Fig. 3, C and D, and fig. S11), which can be attributed to the extra volume and the flexibility of the introduced hydroxypropyl groups that can interfere with the configuration of the HP-γ-CD cavity. These hydroxypropyl groups also modify the host-guest interactions with PFOA and PFOS, producing distinguishable level 2 signals for each PFAS (Fig. 3, E and F, and figs. S12 and S13). Notably, HP-γ-CD–PFOS interaction shows a lower level 2 signal frequency than HP-γ-CD–PFOA, indicating unique spatial interactions between HP-γ-CD and distinct molecular structures of PFOA and PFOS facilitated by hydroxypropyl groups.

Further quantitative analysis of these host-guest interactions was conducted through statistical evaluation of blockade and dwell time distributions (Fig. 3, G and H). The addition of PFOA and PFOS notably increased the level 1 dwell time of HP-γ-CD–PFOA and HP-γ-CD–PFOS to 16.9 ± 1.230 ms and 29.27 ± 3.563 ms, respectively, compared to 0.313 ± 0.018 ms of HP-γ-CD alone. Meanwhile, the level 1 blockade decreased for both to 0.712 ± 0.091 (HP-γ-CD–PFOA) and 0.667 ± 0.114 (HP-γ-CD–PFOS), compared 0.863 ± 0.002 of HP-γ-CD. This reduction in blockade may be due to the additional negative charge of the complexes introduced by PFOA and PFOS, which repels negatively charged amino acid residues (such as Asp or Glu) within the nanopore, reducing spatial obstacles and facilitating ion flow, thereby decreasing the current blockade. In terms of level 2 signals, the blockades were 0.898 ± 0.124 for HP-γ-CD–PFOA and 0.806 ± 0.079 for HP-γ-CD–PFOS, with respective dwell times of 0.087 ± 0.008 ms and 0.087 ± 0.001 ms, indicating distinctive interactions. In agreement with our initial hypothesis, the hydroxypropyl groups on HP-γ-CD markedly prolong the interaction between CD and PFAS molecules and lead to extended dwell times (Fig. 3I). This enhancement facilitates more pronounced differentiation between level 2 signals of PFOA and PFOS molecules while maintaining constant and stable level 1 currents (Fig. 3J). On average, γ-CD– and HP-γ-CD–engineered nanopores displayed 2.3 and 11.4% difference in level 2 relative blockades between PFOA and PFOS, respectively. Notably, the appearance of PFAS signals (level 2) is dependent on the capture of CD molecules (level 1). In independent nanopore tests, we observed that both HP-γ-CD–PFOA and HP-γ-CD–PFOS systems exhibited a linear increase in level 2 and level 1 signals over time (Fig. 3K; the “all HP-γ-CD” events represent the total number of level 1 events for both individual CD and HP-γ-CD–PFOA, the “HP-γ-CD with PFOA” events represent the total number of level 1 events accompanied by level 2 signals, and the “PFOA” events represent the total number of level 2 events over time), which allows us to characterize molecular capture rates through frequencies of various types of events for concentration calculation.

Next, the kinetics of HP-γ-CD adsorption to PFOA and PFOS and their interaction within a nanopore were analyzed through real-time monitoring of current event frequencies. Variations of signal frequencies were recorded for HP-γ-CD incubated with PFOA or PFOS for different amounts of time. An event without multilevel signals was classified as a level 1 only signal, and an event with multilevel signals was classified as a level 2 signal. The appearance of characteristic multilevel signals indicates interactions between HP-γ-CD and PFAS, which marks a decrease in level 1 frequency and an increase in level 2 signals over time. As shown in Fig. 3L, initially, level 1 signal frequency was 582.5/min with no level 2 signal observed. Upon addition of PFOA or PFOS, level 1 signal frequencies decreased, while level 2 signal frequencies increased over time. At the initial stage after PFOA or PFOS addition, unpaired PFAS molecules still disrupted the lipid membrane. The membrane’s stability improved after 2 hours of incubation, indicating PFAS adsorption by HP-γ-CD over time. After 4 hours of incubation, both level 1 and 2 frequencies reached equilibrium. Specifically, frequencies of level 2 events for HP-γ-CD–PFOA and HP-γ-CD–PFOS reached 161/min and 21.5/min after 12 hours of incubation.

### PFOA and PFOS detection sensitivity and specificity

**Fig. 4.**
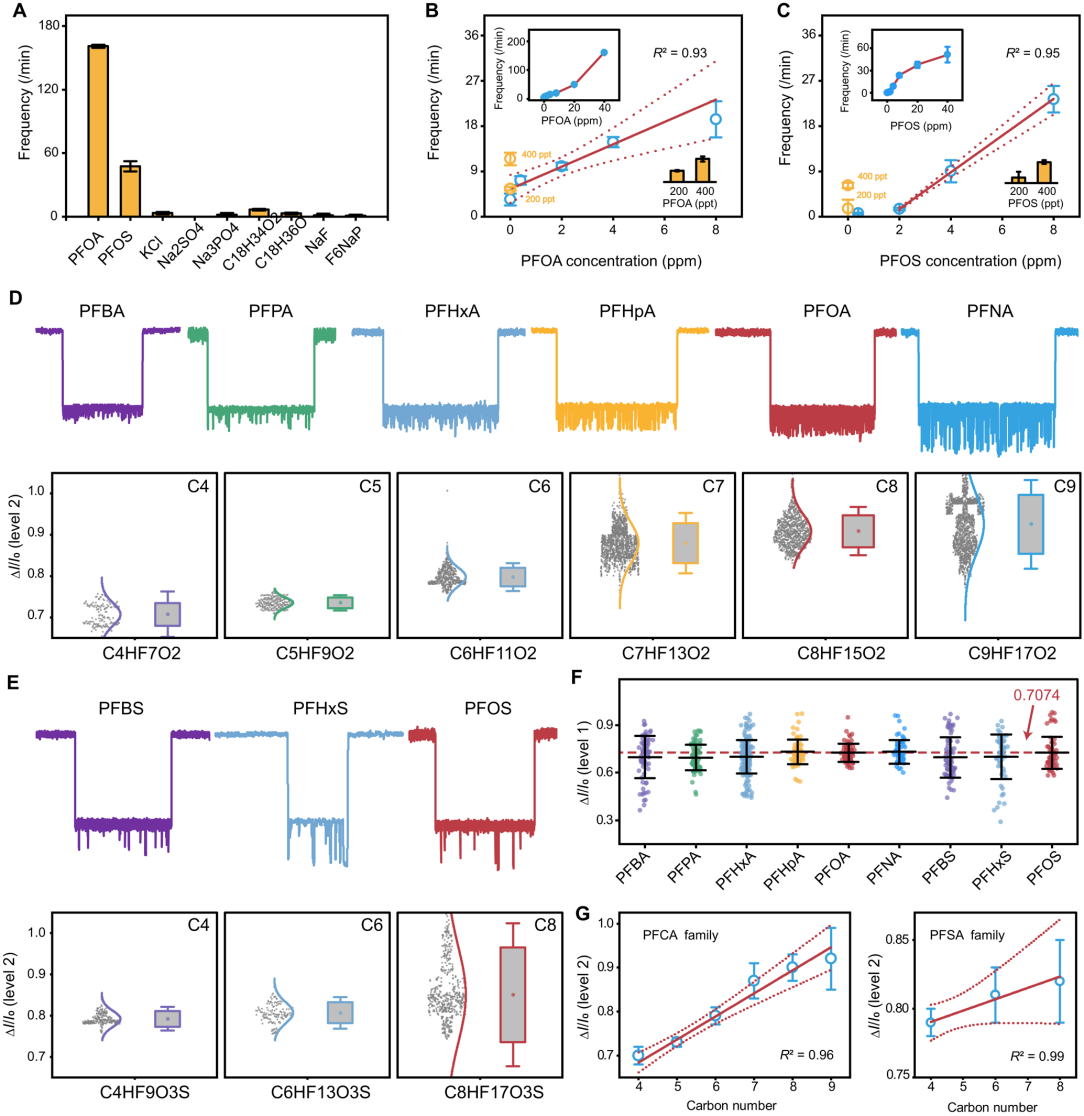
Sensitivity and specificity of HP-γ-CD–mediated PFAS detection and identification by α-HL nanopore. (**A**) Specificity investigation of HP-γ-CD–engineered nanopore by assessing level 2 signal frequencies of different guest substances. (**B** and **C**) Correlation between host-guest interaction induced level 2 signal frequency and the concentration of (B) PFOA and (C) PFOS within 0- to 8-ppm range in water. Insets show correlations within 0- to 40-ppm range. Data represent mean ± SD of replicates (*n* = 3 statistically independent experiments). (**D**) Profiling of PFCA family through HP-γ-CD–mediated nanopore sensing. Top: Representative current traces induced by interactions between HP-γ-CD and different PFCAs. Bottom: Corresponding level 2 blockade distributions. The solid curves show Gaussian fits of blockade distributions. Box plots represent the mean blockade (dot) for all valid signals. The whisker error bars represent 1.5 times the SD of the data. (**E**) Profiling of PFSA family through HP-γ-CD–mediated nanopore sensing. Top: Representative current traces induced by interactions between HP-γ-CD and different PFSAs. Bottom: Corresponding level 2 blockade distributions. The solid curves show Gaussian fits of blockade distributions. Box plots represent the mean blockade (dot) for all valid signals. The whisker error bars represent 1.5 times the SD of the data. Events in (D) and (E) were acquired from nine independent measurements. (**F**) Scatterplot showing average level 1 blockade (Δ*I/I*_0_) for each PFAS sample, with lines representing the mean baseline ± SD across all runs. (**G**) Correlation between mean relative level 2 blockade and molecule chain length for the PFCA and the PFSA families. Data represent mean ± SD of all valid signals. The red solid line is obtained by a linear fit of the mean values (blue circles). The red dotted lines indicate limits of the 95% confidence interval of the fitted line.

In evaluating HP-γ-CD–assisted nanopore sensing for PFOA and PFOS in real-world scenarios, we first tested the system against high salt environments and other common contamination substances, given their typical high concentrations in contaminated water. We focused on the frequency of level 2 signals as the main criterion (Fig. 4A). Despite challenging conditions with 1000-fold excess of various salts, such as KCl (fig. S14), Na_2_SO_4_ (fig. S15), and Na_3_PO_4_ (fig. S16), the system demonstrated remarkable selectivity for PFAS by maintaining notably lower level 2 signal frequencies for salts. This robust specificity was further affirmed as common surfactants such as oleic acid (C_18_H_34_O_2_; fig. S17) and octadecenol (C_18_H_36_O; fig. S18) as well as fluorine-containing compounds (e.g., NaF and F_6_NaP; figs. S19 and S20) also did not show noteworthy secondary signal frequencies that may interfere with PFOA and PFOS detection.

The sensitivity of this system was gauged by the consistent increase in level 2 signals correlating with concentrations of PFOA and PFOS. The correlation exhibits a linear trend at the lower concentration range, allowing for the establishment of calibration curves and limit of detection (LOD) determination: 0.4 ppm for PFOA (Fig. 4B and fig. S21) and 2 ppm for PFOS (Fig. 4C and fig. S22). For detection in regular drinking water with lower concentrations of PFAS, our assay can be effectively used in conjunction with sample preconcentration by rotary evaporation to reach LODs as low as 400 ppt for both PFOA and PFOS [insets in Fig. 4 (B and C) and figs. S23 and S24], despite a mass loss of approximately 20 to 40% during the concentration process (fig. S25). This result aligns with the LOD values obtained through mass spectrometry used as a reference test in this study (fig. S26). Now, high-performance liquid chromatography–tandem mass spectrometry is the gold standard for PFAS detection, yet recent developments have introduced various methods, including optical and electrochemical sensors. We have compared LOD, concentration range, cost, portability, and fabrication complexity between existing techniques and our nanopore-based single-molecule detection method as an emerging frontier (table S2) (*1*, *16*, *18*, *19*, *55*–*63*).

### Identification of homologs of PFCA and PFSA families with different carbon chain length

Above results indicate that HP-γ-CD–mediated host-guest interactions within an α-HL nanopore can afford highly sensitive and specific quantitative sensing of PFOA and PFOS at the single-molecule level. To explore the capability of our approach for distinguishing similar molecules of the diverse PFAS family, we tested PFAS molecules of different chain lengths. We specifically examined members of the PFCA family [heptafluorobutyric acid (PFBA), perfluoropentanoic acid (PFPA), perfluorohexanoic acid (PFHxA), perfluoroheptanoic acid (PFHpA), PFOA, and perfluorononanoic acid (PFNA)] and the PFSA family [PFBS, perfluorohexanesulphonic acid (PFHxS), and perfluorooctanesulfonate (PFOS)], representing carbon chain lengths from C4 to C9 and C4 to C8, respectively. Characterization of level 2 current blockade induced by the host-guest interaction of HP-γ-CD and each type of PFAS in an α-HL nanopore (Fig. 4, D and E, and figs. S27 to S33) and contour plots of level 2 current blockade versus dwell time (figs. S34 and S35) reveals a direct correlation between current blockade ratio and carbon chain length. Notably, for both PFCA (Fig. 4D) and PFSA families (Fig. 4E), longer chains by one to two carbons correspond to increased blockade, underscoring our method’s sensitivity to minor structural variations within the same family.

Quantitative analysis of the level 2 blockade-chain length correlation was enabled by the consistent level 1 blockade as the baseline to allow for accurate comparison across various PFOA or PFOS molecules (Fig. 4F). We observed linear increases in secondary level signal blockade with increasing carbon chain lengths in both PFOA and PFOS families. Specifically, for PFOA, level 2 blockades ranged from 0.707 ± 0.027 (PFBA, C4) to 0.925 ± 0.071 (PFNA, C9); for PFOS, from 0.792 ± 0.019 (PFBS, C4) to 0.820 ± 0.03 (PFOS, C8) (Fig. 4G). Perfluorodecanoic acid (PFDA; C10) exhibited an almost complete blockade (fig. S36), suggesting sensing limitations beyond certain molecular size. Besides the traditional volume exclusion model, these differences in current blockade among various PFAS molecules may also result from noncovalent interactions with CD molecules, influencing solution conductivity within the nanopore and contributing to variations in current blockage (*64*).

Above findings indicate that, while precise identification of PFAS molecules within a suitable range of chain length is feasible using HP-γ-CD as an adaptor in an α-HL nanopore, this has only been achieved in homogeneous samples. For complex mixtures, further development of data analysis algorithms and enhanced sensitivity are needed for quantification of specific species through signal deconvolution. In addition, we have evaluated our current strategy for quantifying a 1:1:1 mixture of PFHxS, GenX, and PFNA. The results show that the LOD for direct detection in the sample is 0.4 ppm, similar to that of PFOA. However, at higher concentrations, the signal frequency is notably higher than that of homogeneous samples, suggesting that interactions between different PFAS molecules in the mixture may contribute to level 2 signal formation (fig. S37).

### Association constant effect on the host-guest interaction

**Fig. 5.**
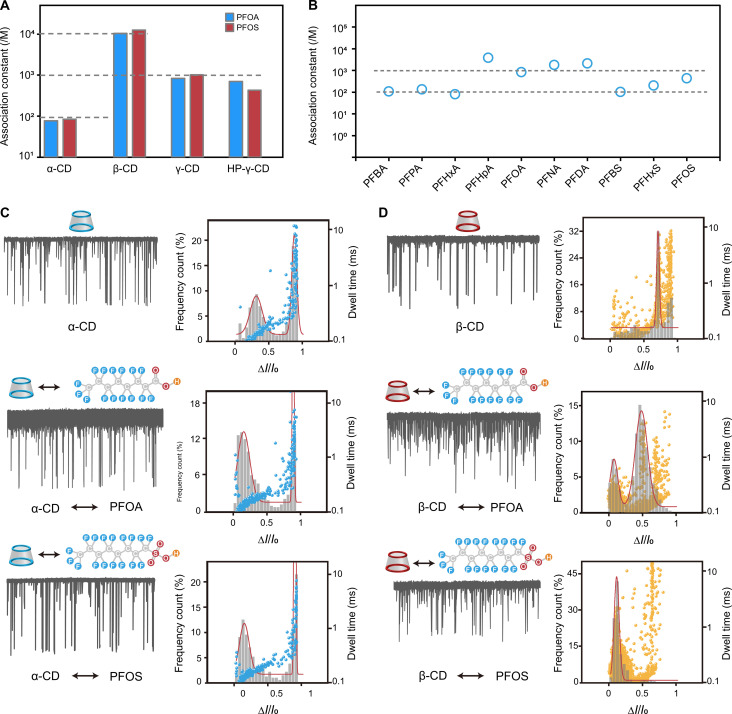
Effects of association constant on interactions between PFAS molecules and CDs within an α-HL nanopore. (**A**) Association constants of PFOA and PFOS with different CDs including α-CD, β-CD, γ-CD, and HP-γ-CD. (**B**) Association constants of different molecules of the PFCA and the PFSA families with HP-γ-CD. (**C**) Profiling of interactions between α-CD and PFAS molecules within an α-HL nanopore. Left: Continuous raw current traces of α-CD, α-CD-PFOA, and α-CD-PFOS, respectively. Right: Corresponding two-dimensional scatterplots of relative blockade versus dwell time of valid events from nanopore results and histograms of normalized event frequency versus current blockade. (**D**) Profiling of interaction between β-CD and PFAS molecules within α-HL nanopore. Left: Continuous raw current traces of β-CD, β-CD-PFOA, and β-CD-PFOS, respectively. Right: Corresponding two-dimensional scatterplots of relative blockade versus dwell time of valid events from nanopore results and histograms of normalized event frequency versus current blockade.

Previous studies have shown that CDs, especially β-CD, are effective in complexing with PFAS molecules (*3*, *65*), hinting at their potential for PFAS contamination remediation (*66*). The host-guest complex formation is driven by van der Waals interaction between CD’s hydrophobic cavity and PFAS’s aliphatic backbone (*67*, *68*). To delve deeper into the possible chemical mechanisms underlying our observations, we analyzed the ^19^F NMR spectra of PFAS molecules involved with varying CD concentrations. By analyzing the nonlinear least-squares regression of observed chemical shift changes (figs. S38 to S45), association constants between various CDs and PFAS were determined (table S3). Magnitudes of association constants between PFOA or PFOS and different CDs are shown in Fig. 5A. On the basis of our findings, α-CD shows weak or nonexistent complexation with PFOA or PFOS, indicated by (10^1^ to 10^2^) M^−1^ association constants. In contrast, β-CD demonstrated the strongest interactions with both PFOA and PFOS, evidenced by its notably higher association constants of (10^4^ to 10^5^) M^−1^. γ-CD exhibited moderate association constants in the range of (10^2^ to 10^3^) M^−1^. These results align with prior research indicating a hierarchy of β-CD > γ-CD > α-CD in terms of binding strength (*67*, *69*), attributed to how well the cross-sectional area of PFAS matches with each type of CD (*70*). Notably, γ-CD and HP-γ-CD both exhibited a similar, moderate level of association with PFOA and PFOS. For molecules of different carbon chain lengths within the PFCA and PFSA families, association constants with HP-γ-CD remained consistently moderate (10^2^ to 10^3^) M^−1^, albeit slightly influenced by the number of ─CF_2_─ groups (Fig. 5B). Such moderate binding affinity should allow the formation of “loose” complexes between PFAS and γ-CD or HP-γ-CD, inducing current blockage signals from CD molecules driven by electroosmotic flow. Concurrently, PFAS molecules alter this process through host-guest interactions, leading to the creation of a unique “fingerprint” signal for each type of PFAS based on the CD blockage signal.

To validate our speculation, we substituted γ-CD and HP-γ-CD with α-CD and β-CD, known for their weakest and strongest interactions with PFOA and PFOS, respectively. Their mismatched binding affinity with PFAS molecules resulted in complexes that are incapable of generating distinctive level 2 signals in the nanopore system. α-CD maintained two level 1 blockade subpopulations at ~0.3 and ~0.9 before and after the addition of PFOA or PFOS (Fig. 5C, fig. S46, and table S4). In contrast, β-CD, because of its strong affinity with PFOA or PFOS, presented clear blockade distribution changes upon interaction (Fig. 5D, fig. S47, and table S4), even distinguishable when measuring a mixture of PFOA and PFOS using β-CD (fig. S48). These findings reinforce the importance of selecting CD with appropriate binding affinity for PFAS detection.

### Ion transport modeling

Using a three-dimensional finite element method (FEM) model and Poisson-Nernst-Planck (PNP) equations, we derived detailed local profiles of ion flux and electric potential to understand the molecular mechanisms behind the observed pore conductance values. Our model simulated ion diffusion and migration under an applied potential to help interpret the blockade ratios of various molecules. The ion channel geometry was set up according to the α-HL protein structure (fig. S49A). The finite size of the ions was taken into account by considering their distance of maximum approach to molecular surfaces (i.e., we considered an exclusion zone for the ions next to all molecular surfaces with a thickness equal to the radius of the hydrated ions, 0.2 nm). Then, the local electrostatic potential and ion flux in the nanopore with and without various molecules in the cavity were calculated under a 100-mV bias (fig. S49, B and C). The predicted net ion current for an open α-HL (305 pA) closely approximates the experimental measure (268 pA). CDs were described as cylindrical shells located at the base of the nanopore cavity. Table S5 summarizes dimensions, currents, and blockade ratios for α-CD, β-CD, and γ-CD. The model’s blockade ratio predictions align well with empirical data, showing errors of 0, 16.6, and 3.0% for α-CD, β-CD, and γ-CD, respectively. These results support the proposed geometry of CDs and α-HL systems. For the less reported HP-γ-CD, we fine-tuned the inner diameter according to experimental blockade ratios, resulting in a 0.15-nm reduction compared to γ-CD to account for the structural impact of the 2-hydroxypropyl groups. PFOA and PFOS were characterized as rigid, cylinder-like molecules with a 0.5-nm diameter located at the pore’s constriction and were examined in two spatial configurations within the nanopore: aligned with the pore axis (on-axis) and adjacent to the pore wall (off-axis). The on-axis position resulted in a larger effective cross section than the off-axis position because of the ion exclusion zone around the molecule. Consequently, the off-axis configuration exhibited a lower blockade ratio of 0.15, more closely matching experimental measurements of ~0.12. This suggests that PFOA and PFOS predominantly translocate near the pore wall, offering insights into the MD within the nanopore environment.

### MD simulations

**Fig. 6.**
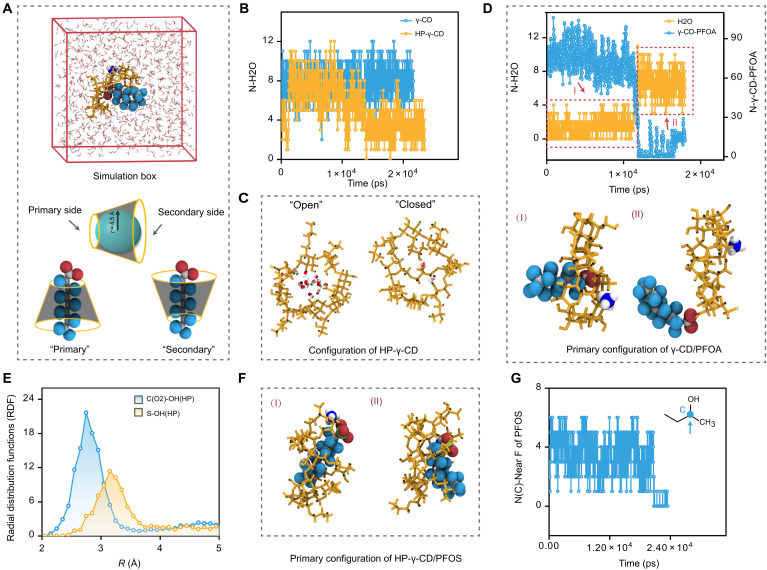
Molecular simulation of interactions between CDs and PFAS molecules. (**A**) Top: Schematic illustration of the simulation box containing H_2_O, CD, and PFAS molecule. Bottom: Schematic illustration of CD identifying its primary and secondary sides and the sphere of 4.5-Å radius inside the CD in which the number of H_2_O molecules is monitored. The two bottom schemes illustrate two initial configurations for each system: In both cases, the PFAS was initially inserted inside the CD but in one case with the head group oriented toward the primary side of the CD and in the other the secondary side. (**B**) Time evolution of instantaneous numbers of H_2_O molecules in the 4.5-Å sphere inside γ-CD and HP-γ-CD in pure water (without PFAS molecules). (**C**) Two representative snapshots of HP-γ-CD in pure water with water molecules inside the inner cavity. (**D**) Top: Time evolution of instantaneous number of H_2_O molecules inside γ-CD inner cavity and number of contacts between γ-CD and PFOA atoms (within 4.0-Å distance). Bottom: Corresponding snapshots of γ-CD-PFOA with the primary configuration at the beginning (I) and the end (II) of the trajectory. (**E**) Radial distribution functions between the C atom of the PFOA head group and the OH group of the HP tether in HP-γ-CD, as well as the S atom of the PFOS head group and the OH group of the HP tether in HP-γ-CD. (**F**) Snapshots illustrating HP-γ-CD–PFOS in configurations where PFOS interacts with four to five HP tethers simultaneously. (**G**) Time evolution of the number HP groups (defined by selected C atoms of HP tethers) and F atoms of PFOS in HP-γ-CD–PFOS system.

To elucidate the HP-γ-CD–assisted nanopore sensing mechanism for PFAS, we conducted all-atom MD simulations. Previous studies have revealed that both γ-CD and HP-γ-CD can align their eightfold molecular axis with the sevenfold axis of the heptameric α-HL pore (*49*). CD insertion into the nanopore redirects ion transport through its inner cavity, enhancing the sensitivity to the host-guest interaction between the CD’s hydrophobic cavity and PFAS. This process displaces water molecules from the CD cavity, alters ion transport channels, and causes varying ion current blockages, as observed in our experiments. The role of water molecules is crucial in this simulation as demonstrated in a previous study, in which the number of water molecules in the *Mycobacterium smegmatis* porin A (MspA) nanopore system has a strong correlation with ionic current blockade to afford DNA sequence specificity (*71*). Our detailed investigation through MD simulations of CD/PFAS/NH_4_ complexes in water provides insights into the mechanisms. In a representative simulation snapshot, we observed dual configurations for PFAS entering the CD cavity, determined by whether the PFAS head group faces the CD’s primary or secondary side (Fig. 6A). The primary configuration, in which the head group faces the primary side, appears more prevalent due to CD’s orientation in the nanopore and PFAS’s directional diffusion. We analyzed the CD cavity’s internal environment using a 4.5-Å radius sphere centered at the CD’s mass center, focusing on the water molecule presence. This approach highlights the role of water in facilitating ion movement with minimal disruption to their hydration shell. A lack of sufficient water creates a challenging environment for ions, forcing them to dehydrate and navigate past other species. This suggests that a crucial balance in the CD cavity’s hydration is essential for effective ion transport. Figure 6B illustrates the variability of water molecules within the 4.5-Å radius sphere in CD’s cavity in the absence of PFAS. γ-CD generally contains 5 to 12 water molecules, with an average of around 8. Contrastingly, HP-γ-CD displays a bimodal distribution, fluctuating between numbers akin to γ-CD and considerably lower counts (1 to 5), as seen in fig. S50A. We categorize these HP-γ-CD states as “open” and “closed” (Fig. 6C). In the closed state, few water molecules are present, creating a hydrophobic environment that impedes ion transport. This aligns with experimental results where HP-γ-CD shows a higher level 1 blockade ratio (0.863) compared to γ-CD (0.674). Both simulation and experimental results suggest that HP-γ-CD presents a more notable barrier to ion passage due to its flexible hydroxypropyl groups obstructing the pore.

We next explored PFAS complexation with CDs by guiding the PFAS tail into the CD cavity and orienting the head group toward either the primary or secondary side. Our study identified two distinct configurations for γ-CD and PFOA, evident in the variation of water molecules and the number of close contacts (less than 4.0 Å) between them (Fig. 6D). In the first configuration (snapshot “I”), inserting the PFOA tail into the γ-CD cavity notably reduces water content. The remaining water molecules are unable to maintain their usual hydrogen bonding network, thus interact with PFOA’s fluorinated tail instead and create an atypical and stressed environment. Despite the constrained environment, this configuration remained stable over multiple nanoseconds, suggesting low free energy. In the second configuration (snapshot “II”), the PFOA tail vacates the γ-CD cavity and is replaced by more water molecules, while the PFOA head group continues strong interactions with CD’s polar groups (e.g., OH groups) and the fluorinated tail interacts with CD’s hydrophobic outer side. In the first configuration, with the PFOA tail inserted, little to no additional ion transport through the CD is expected, leading to increased blockade and level 2 signals in experiments. Conversely, when the tail is outside the γ-CD cavity (second configuration), the current blockade resembles that of γ-CD alone, reverting to level 1 intensity. The alternation between these configurations likely explains the multiple level 2 events superimposed on a single level 1 event. Simulations with PFOS showed a similar trend and mechanism (fig. S50B), indicating that, during complexation of γ-CD with PFOA or PFOS, the fluorinated tail does not always locate inside the inner cavity of CD and blocking it for water and ion transport. Rather, the PFOA or PFOS molecule adjusts its electrostatic interactions with γ-CD and the surrounding environment, as various configurations permit contact between the fluorinated tail and γ-CD’s hydrophobic parts.

In HP-γ-CD, the PFAS association is further complicated by HP tethers. PFOA is more hydrophilic than PFOS, demonstrated by its nearly five times higher critical micelle concentration in water. PFOS’s additional CF_2_ group increases its hydrophobicity and disperses its charge across the ionic head group, resulting in weaker electrostatic interactions with the OH groups of HP tethers compared to PFOA. This is corroborated by the radial distribution functions shown in Fig. 6E, where PFOS demonstrates weaker electrostatic interactions with HP tethers. To compensate, PFOS adjusts its orientation for better hydrophobic contact with HP tethers and CD, at times engaging with four to five HP tethers simultaneously (Fig. 6F). A time evolution study of HP tethers interacting with PFOS (Fig. 6G) reveals a stable configuration of PFOS alternating between inside and outside the CD cavity. This configuration, while stable, is rare due to entropic disadvantages imposed by HP-γ-CD conformations, aligning with the low frequency of level 2 events observed for HP-γ-CD–PFOS complexes. In contrast, HP-γ-CD–PFOA complexation relies less on multiple HP tethers, with PFOA forming stronger electrostatic interactions and adjusting its orientation for enhanced hydrophobic contact with CD. This leads to transient blockages and a higher frequency of level 2 events, as observed in experiments. Our simulations indicate that while PFOS forms more stable but infrequent configurations, PFOA adapts more dynamically to induce higher frequency of ion blockages. The time evolution of water molecule numbers also confirms this trend (fig. S50, C to G). Notably, while the above MD simulation results further elucidate the interactions between CD and PFAS and reflect the corresponding nanopore signals, the current simulation system has not yet incorporated the protein pore structure. With further improvements by adopting the more sensitive steric exclusion model (*72*), we will attempt to calculate the theoretical current blockage values of the PFAS molecules.

## DISCUSSION

PFAS, widely used in chemical engineering industries, are compounds with varying fluorinated carbon chains and functional groups (*73*). Because of their robust structures, PFAS cause long-lasting contamination to water resources and environmental health crisis in many regions. Exposure to PFAS has been linked to cancers, especially kidney and testicular cancers, and endocrine disruption affecting thyroid hormones (*74*). These substances can impair reproductive health, leading to reduced fertility and adverse fetal development. PFAS exposure also weakens the immune system, reduces vaccine efficacy, and causes metabolic issues like elevated cholesterol and liver damage. In addition, PFAS are associated with hypertension and declined kidney function. Their persistent and bioaccumulative nature further amplifies these risks, highlighting the need for stringent regulation and ongoing research (*75*).

Efficient detection methods for PFAS in water are vital for reducing exposure and facilitating remediation and water reuse. Traditional PFAS detection relies on chromatography and mass spectrometry, requiring centralized facilities, skilled operators, and extensive preparation, despite offering high accuracy and sensitivity. Recent advances aim to simplify PFAS detection using nanoscale materials and photochemical or electrochemical methods to reduce costs and complexity (*17*, *76*). Yet, challenges in sensitivity and selectivity persist, underscoring the need for enhanced sensor-PFAS interactions for improved detection. Drawing on the unique affinity of CD molecules for PFAS and the size compatibility with α-HL nanopore, we explored sensing the most common PFAS molecules: PFOA and PFOS, through CD-assisted nanopore technology, which is increasingly capable of detecting smaller entities like DNA and proteins (*77*–*79*). Our investigations into host-guest interactions of PFOA or PFOS with α-CD, β-CD, γ-CD, or HP-γ-CD within α-HL nanopore revealed that the association constant between CD and PFAS is crucial for single-molecule sensing. Specifically, α-CD’s weak binding to PFAS cannot afford the formation of a host-guest complex, while β-CD’s strong affinity prevented PFAS disengagement from the complex, both hindering PFAS-specific level 2 signal generation within a nanopore. PFOA and PFOS sensing was achieved with γ-CD due to its moderate binding affinity, and further discrimination of various members of PFCA and PFSA families at the molecular level was achieved with increased steric resistance of HP-γ-CD, enhancing both selectivity and sensitivity. Our results show that this CD-assisted nanopore sensing strategy can detect PFOA and PFOS at the ppt level with sample preconcentration and is sensitive to the carbon chain length of a PFAS molecule within a certain range. This ability to differentiate various homologs of PFCA and PFSA molecules highlights the method’s potential as a robust tool for PFAS analysis.

Over the past years, β-CD supramolecular macrocycles have been used to refine α-HL nanopore’s ability to differentiate nucleotides and organic compounds by fitting into the lumen of modified α-HL proteins, followed by introducing organics for sensing (*80*, *81*). However, such methods often overlooked the impact of organic-lipid membrane interactions on detection sensitivity, especially for lipophilic substances like PFAS. Given their high lipophilicity and associated bioaccumulation risks (*82*), naked PFAS translocation through nanopores is hindered because they cause lipid membrane disturbances (*83*). In contrast, our approach uses CDs not only as adaptors but also as adsorbents within the nanopore system. By encapsulating PFAS within the hydrophobic cavities of CDs, our strategy isolates them from the lipid membrane that supports the nanopore and also leverages the cavities as narrow channels to enhance nanopore detection sensitivity and specificity for PFAS. These findings suggest more potential applications of modified CDs or other supramolecular macrocycles as adaptors in nanopore-based sensing.

Nonetheless, while this proof-of-concept study presents opportunities for practical applications, further developments are still needed to address several challenges and limitations. First, confirmation of the dissociation of a PFAS molecule from a CD and its subsequent translocation through the nanopore to experimentally validate the mechanism of level 2 signal generation is yet to be achieved. This challenge arises from the instability of lipid membranes upon interacting with PFAS, which hinders the collection of adequate PFAS molecules at the trans side of the nanopore for measurement by a reference assay. To overcome this, developing hybrid nanopore (*84*, *85*) or replacing liposomal membranes with polymer membranes (*86*, *87*) could be effective. These strategies would not only reduce or eliminate the PFAS-CD preincubation time before measurement but also tackle the stability issue for commercialization of this technology. Second, the LOD of the current method does not yet meet the stringent requirements for low concentration PFAS detection. Despite LOD improvement from 0.4 to 2 ppm to 400 ppt by sample preconcentration, there remains a substantial discrepancy with current EPA standards. Potential approaches for sensitivity enhancement include modifying CDs by introducing groups such as silane or amine that have a strong affinity for fluorine to increase PFAS adsorption. In addition, from the nanopore instrumentation perspective, increasing sampling frequency and optimizing the work solution and detection voltage are all promising avenues to explore. Third, the current method has effectively differentiated several common PFAS compounds by carbon chain length and demonstrated detection of a PFAS mixture. Despite these advancements, the stochastic nature of nanopore sensing makes identifying specific PFAS species from mixture samples challenging. The abovementioned sensitivity improvement strategies may help us gather more information of PFAS molecules from their signals, such as fluctuation of current events (*88*, *89*), to accurately distinguish specific species through signal deconvolution. Moreover, we are exploring machine learning algorithms to establish a comprehensive fingerprint database of PFAS for data analysis of unknown mixture samples. Last, considering the scalability for commercialization of this platform technology, several commercial nanopore products for gene sequencing (e.g., Oxford Nanopore MinION, QitanTech Nanopore) provide valuable development models. However, for a device aimed at on-site testing of contaminated water, the nanopore reader must be integrated with miniaturized sample pretreatment equipment for processing complex environmental water to ensure detection reliability. In addition, automated device control and signal processing are essential for meeting real-world demands, particularly in resource-limited settings.

Addressing the abovementioned issues will markedly bolster the capacity and the applicability of our CD-mediated PFAS sensing strategy. The resultant system will be particularly suited for field deployment and in-lab analysis, leveraging the swift progress and the widespread availability of nanopore technology and devices. With these promises, our sensing approach stands to substantially advance PFAS monitoring and detection, delivering rapid, accessible, affordable, and portable solutions for both routine and urgent environmental testing needs.

## MATERIALS AND METHODS

### Materials

PFAS including PFBA (C_4_HF_7_O_2_), PFPA (C_5_HF_9_O_2_), PFHxA (C_6_HF_11_O_2_), PFHpA (C_7_HF_13_O_2_), PFOA (C_8_HF_15_O_2_), PFNA (C_9_HF_17_O_2_), PFDA (C_10_HF_19_O_2_), PFBS (C_4_HF_9_O_3_S), PFHxS (C_6_HF_13_O_3_S), and PFOS (C_8_HF_17_O_3_S) were purchased from SynQuest Laboratories. CDs, including α-CD, β-CD, γ-CD, and HP-γ-CD, as well as tris hydrochloride (tris-HCl), deuterium oxide (D_2_O, 99.9%), α-HL from *Staphylococcus aureus* (lyophilized powder, protein ~60% by Lowry, ≥10,000 U/mg protein), *n*-pentane (anhydrous, ≥99%), and hexadecane (Reagent Plus, 99%), were purchased from Sigma-Aldrich. Lipids containing 1,2-diphytanoyl-*sn*-glycero-3-phosphocholine (4ME 16:0 PC) were purchased from Avanti Polar Lipids (Alabama, USA). All chemicals were used without further purification. All work solutions were prepared using deionized (DI) water from a Milli-Q water purification system (resistivity 18.2 megohm·cm, 25°C; Millipore Corporation) and were filtered through 0.02-μm filters before use.

### Sample preparation

#### Preparation of PFAS stock solution

Each PFAS sample was dissolved in DI water to prepare an individual stock solution with a concentration of 10 mg/ml. The stock solutions were stored in a refrigerator (4°C) for future use. All PFAS stock solutions were used at room temperature and subjected to necessary ultrasonic treatment or vigorous shaking before use.

#### Preparation of sample solution

All the CD-PFAS mixed solutions were produced by incubating CD and PFAS molecules at room temperature with the molar ratio of 1:1. Taking the HP-γ-CD–PFOA sample as an example, 2.4 × 10^−6^ mol (0.0032 g) of HP-γ-CD powder was dissolved in 400 μl of DI water under ultrasonic condition in a 1.5-ml centrifugation tube. The dissolved solution was mixed with 100 μl of the PFOA stock solution (10 mg/ml) and incubated on a shaker for 12 hours at room temperature (22°C). Last, the sample solution was achieved and stored at 4°C for further tests. To establish a calibration curve for PFOA or PFOS, standard samples were made by adding various volumes of PFOA or PFOS stock solution (10 mg/ml) to DI water containing 2.4 × 10^−6^ mol dissolved HP-γ-CD to form a mixture solution, with a final volume of 500 μl. The mixture solution was vortexed for 12 hours at room temperature (22°C) to complete the standard sample preparation.

### ^19^F NMR spectroscopy

NMR experiments were performed with a 500-MHz Bruker instrument with a quad probe (operating at 376.498 MHz for ^19^F). Hexafluorobenzene was used as the internal standard, with a chemical shift of −164.9 ppm. The chemical shift of each peak was recorded, and the 1:1 PFOA:CD association constants were calculated using the method developed by Cabrer *et al*. (*70*, *90*).

### Single nanopore electrical recording

In our nanopore experiment, two chambers, cis and trans, were separated by a planar lipid bilayer, formed on a 200-μm aperture in a 25-μm-thick Delrin wall (*41*). This bilayer was self-assembled using 1,2-diphytanoyl-*sn*-glycero-3-phosphocholine. Each chamber contained 1 ml of 3 M KCl and 10 mM Tris-HCl (pH 8), and the cis compartment was grounded. Following the decane film’s thinning and the lipid bilayer’s establishment, we applied an electrical potential to the trans side via Ag/AgCl electrodes using a Planar Lipid Bilayer Workstation and incrementally increased it to assess membrane stability between (±100 and ±200) mV. Throughout the experiment, we continuously monitored the lipid membrane’s stability and alterations by recording the membrane capacitance curve with an oscilloscope, maintaining a capacitance of (160 to 180) pF across different voltage biases. This careful monitoring ensured the bilayer’s integrity and stability, critical for the precise measurement of ionic currents through the nanopore. For nanopore insertion, α-HL protein was introduced into the cis side (final concentration, 0.01 to 0.05 ng/ml) under a +100-mV bias, followed by the addition of 20-μl samples. Ionic currents were then recorded at a +100-mV holding potential. To minimize external noise, a Faraday cage on an antivibration surface was used. Electronic signals were captured and amplified with a patch clamp amplifier, filtered at 5 kHz, digitized at 100 kHz (time resolution is 0.01 ms) by a Digidata 1440 A converter, and analyzed with pClamp10 software.

### Data analysis

All events in the nanopore detection data were extracted using an in-house MATLAB-based algorithm (Apperture) and analyzed by Clampfit 10.7 and Origin 2021 software. The extracted data included the blockade and the dwell time of each eligible signal event, which are two commonly used parameters for identifying different analytes. The current blockade (i.e., current drop), representing the capture of a single molecule and its translocation through the nanopore, is defined as ∆*I/I*_0_ (∆*I = I − I*_0_; *I*: the average current blockade measured with the molecules inside the pore; *I*_0_: average baseline value in the absence of analytes). Dwell time (i.e., duration) represents the effective interaction time between the nanopore and a single analyte. Unless otherwise stated, all data were acquired in 3 M KCl and 10 mM tris-HCl buffer, at pH 8.0 and under a +100-mV bias applied to the trans side.

### Data processing and extraction

The data were processed and extracted using an in-house made “Aperture” program. Aperture app was developed by using the basic MATLAB interface and selecting structures initialized in startup code to store data and application components. To facilitate peak extraction, the Aperture app starts by plotting the abf file (current-time data) in the interface. The signal peaks can be identified accurately by setting baseline, peak threshold, and time boundaries through auto-generated settings within a user-friendly graphical interface, which were designed to exclude most baseline noise. After extraction, the data can be saved as an Excel file for detailed analysis.

During extraction, the algorithm marks the initial dip below and rise above the threshold as each peak’s start and end and then calculates the relative current blockade and the dwell time of the peak. Current blockade and dwell time are calculated from the peak start/end values and current data, respectively, with blockade presented as a dimensionless value between 0 and 1, normalized against the baseline. For level 1, if a peak starts and ends at the same point, it is classified as a spike, indicating no measurable dwell time and typically representing noise or transient signals. For level 2 peaks within the level 1 peak boundaries, the extraction function is reapplied, with these often being spikes and not excluded. The baseline value is established by applying MATLAB’s “ischange” function to a smaller dataset (e.g., 1000 points) to divide the data into sections with unique median values. Sections whose medians are similar, determined via wide histogram binning, create a dataset for calculating the baseline. This approach is vital for removing skewed data from overlapping first-level peaks, ensuring the data’s SD accurately represents peak noise. The threshold value is derived by multiplying this SD by a “*z* score,” a statistical metric for baseline confidence level, typically set at 3.5 for a 99.9% confidence level. This accounts for the lower mean values compared to medians, often seen with second-level peaks.

### Ion transport modeling

The *r*(*z*) profile of the α-HL pore was obtained from its crystallographic structure (Protein Data Bank: 7AHL) (*91*). Briefly, water molecules were first deleted from the structure, and then the long axis of α-HL was aligned with the *z* axis. The *r* positions of all atoms in the structure were calculated and plotted, from which the *r*(*z*) profile of the cavity was estimated.

The *r*(*z*) profile of the pore was then used to construct a full three-dimensional FEM model, which was implemented in Comsol Multiphysics 5.2. The pore conductance was determined by solving the PNP equations, which model ion diffusion and migration in the presence of an applied potential bias (*92*, *93*). To account for the fact that the dimensions of the system are commensurable with the size of hydrated ions, an important modification to the previous model was introduced herein to explicitly consider the minimum distance of approach between molecules and the center of the ions. This distance is equal to the radius of the ion, *r*_ion_, (*r*_ion_ = 0.2 nm was used for hydrated K^+^ and Cl^−^), which leads to effective radii of α-HL and CD cavities that are smaller than the real radii (i.e., the values obtained from the molecular structures; see table S5) by the amount of *r*_ion_. In analogy, the effective outer radii for CDs, PFOA, and PFOS used were larger than the molecular radii by the amount of *r*_ion_. Taking into consideration of this exclusion zone for the ions is crucial to properly describe the transport properties of α-HL but would have had an insignificant effect on previous results for larger pores (*93*).

In the FEM model, the same diffusion coefficient (*D*) was used for K^+^ and Cl^−^, which was estimated from the conductivity (κ) of the 3 M KCl solution used in the experiments using the equation

$$D=\frac{\mathrm{RT}}{2F^{2}}\frac{\kappa }{c}$$

where *c* is the concentration of KCl. Last, we neglected the presence of the charges of α-HL and PFOS/PFOA in the calculation because of the very high ionic strength used in the experiments (3 M KCl).

### Molecular dynamics simulations

MD simulations were performed using an in-house simulation package with the nonpolarizable version of Atomistic Polarisable Potential for Liquids, Electrolytes, and Polymers (APPLE&P) force field (*94*, *95*). This force field was found to describe well the aqueous solution of CD and PFAS and predict various properties consistent with experiments (*96*–*100*). The temperature (300 K) and pressure (1 atm) in the simulation box were controlled through the Nose-Hoover thermostat and Anderson-Hoover barostat, respectively (*101*, *102*). The length of all chemical bonds was constrained using the SHAKE algorithm (*103*). A cutoff distance of 15.0 Å was used for the calculation of van der Waals forces and the real part of electrostatic interaction. A multiple time step integrator was used to improve computational efficiency. The shortest time step of 0.5 fs was used for the bonds, bends, and torsions, a medium time step of 1.5 fs was used for the short-range interactions (<8.0 Å), and the largest time step of 3.0 fs was used for the long-range (>8.0 Å) nonbonded interactions and the reciprocal part of Ewald summation.

A three-dimensional periodic simulation box contained a mixture of CD, PFAS (PFOA or PFOS), ammonium counterion (NH_4_^+^), and water. The choice of NH_4_^+^ as the counterion was due to the availability of accurate force field for PFAS/NH_4_^+^ complexation developed within APPLE&P force field. In the investigated solution conditions, the PFAS are mostly dissociated from their counterions, and therefore, their complexation with CD is mostly uninfluenced by the cation. The water model used in our simulation is TIP4P/2005 (*104*). MD simulations were performed with γ-CD and HP-γ-CD. All systems contained one CD molecule, one PFAS molecule, and 960 water molecules. In the initial configuration, PFAS was present inside the CD cavity, while NH_4_^+^ and water molecules were randomly distributed in the box. With PFAS inside the cavity, two different configurations are possible, i.e., “primary” with the headgroup of PFAS pointing toward the primary side and “secondary” with the opposite orientation of PFAS. Therefore, for each system, we investigated two different starting configurations.
