# Supplementary material for: Single-molecule profiling of per- and polyfluoroalkyl substances by cyclodextrin mediated host-guest interactions within a biological nanopore
Source: Sci Adv. 2024 Nov 6;10(45):eadp8134. doi: 10.1126/sciadv.adp8134 (PMC11540018; doi:10.1126/sciadv.adp8134)
Supplement: Supplementary file 1 — Figs. S1 to S50 Tables S1 to S5 [file sciadv.adp8134_sm.pdf]

Science Advances
AAAS

Supplementary Materials for

Single-molecule profiling of per- and polyfluoroalkyl substances by cyclodextrin mediated host-guest interactions within a biological nanopore

Xiaojun Wei *et al.*

Corresponding author: Chang Liu, chang@umass.edu

Sci. Adv. **10**, eadp8134 (2024)
DOI: 10.1126/sciadv.adp8134

This PDF file includes:

Figs. S1 to S50
Tables S1 to S5

Unless otherwise stated, all data was acquired in 3 M KCl, 10 mM Tris-HCl buffer, at pH 8.0 and under a +100 mV bias applied to the *trans* side.

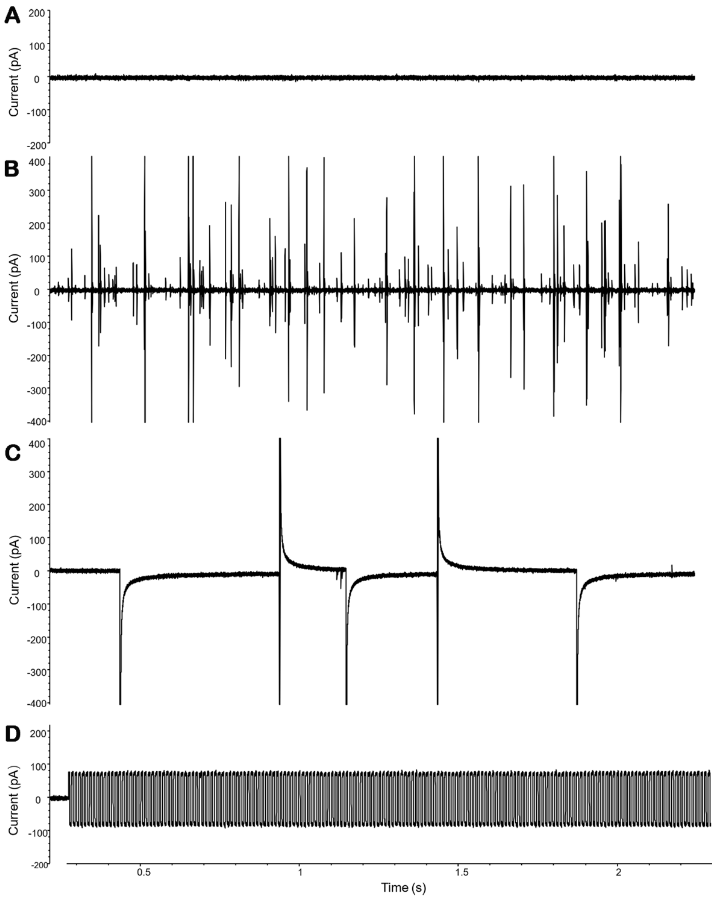

Fig. S1. Stability investigation of the lipid membrane. A: Typical current traces of the lipid membrane without adding α-HL protein in chambers. **B-C:** Current traces reflecting fluctuations of the lipid membrane when (**B**) using a stir bar in the cis chamber or (**C**) flipping voltage between ±200 mV. **D:** Typical current traces of the lipid membrane recorded using capacitance monitor.

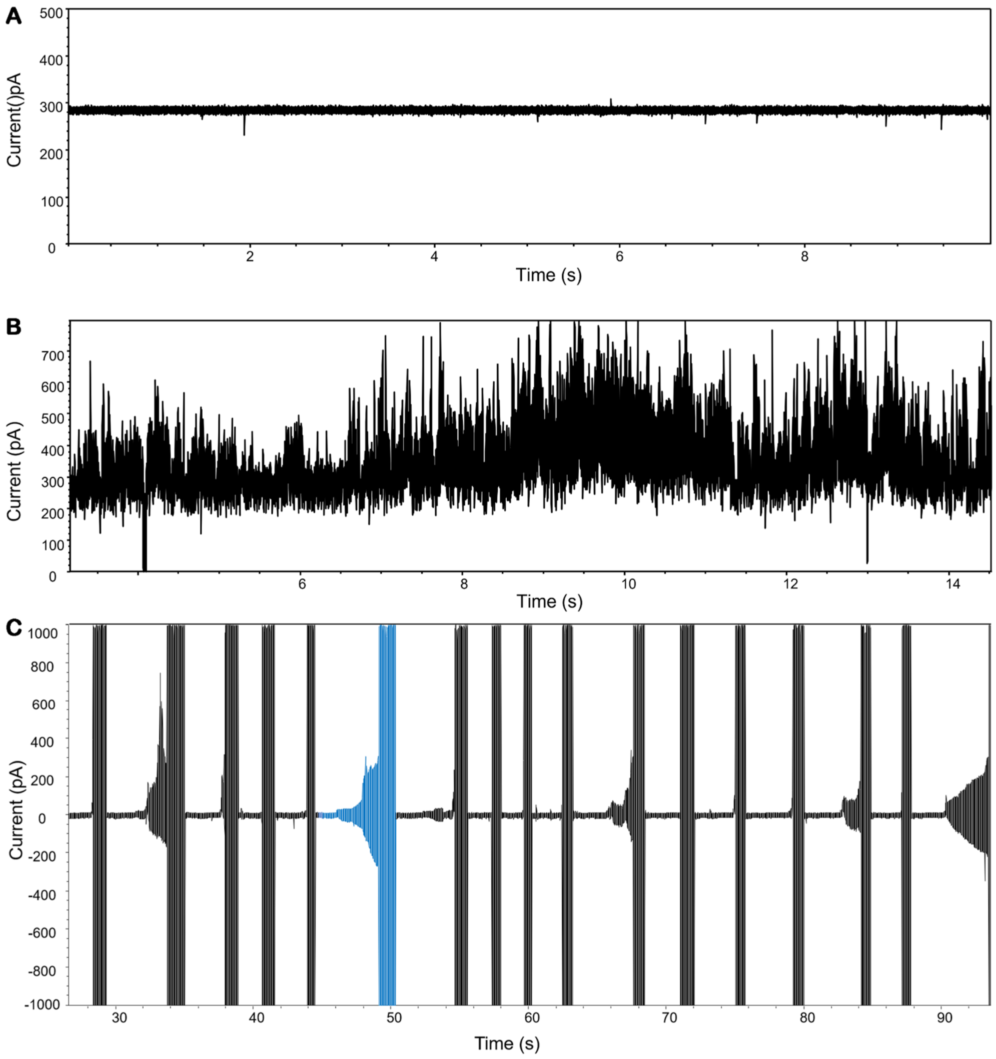

Fig. S2. Current traces and capacitance response of α-HL nanopore and lipid membrane under the influence of PFOA. A: The raw current trace of a blank buffer. **B:** The raw current trace recording of 0.1mM PFOA through α-HL nanopore. **C:** Typical capacitance current trace of a lipid bilayer membrane when PFOA molecules attack the membrane. The blue colored trace was enlarged and shown in Fig. 1D.

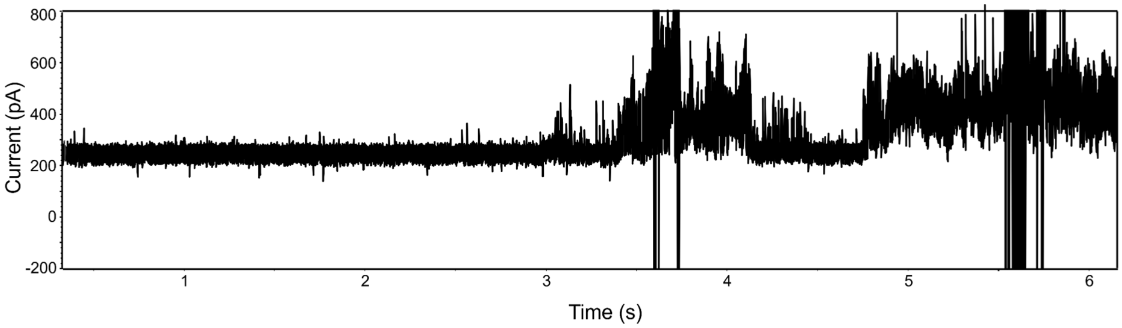

Fig. S3. The raw current trace recording of 0.1mM PFOS through α-HL nanopore.

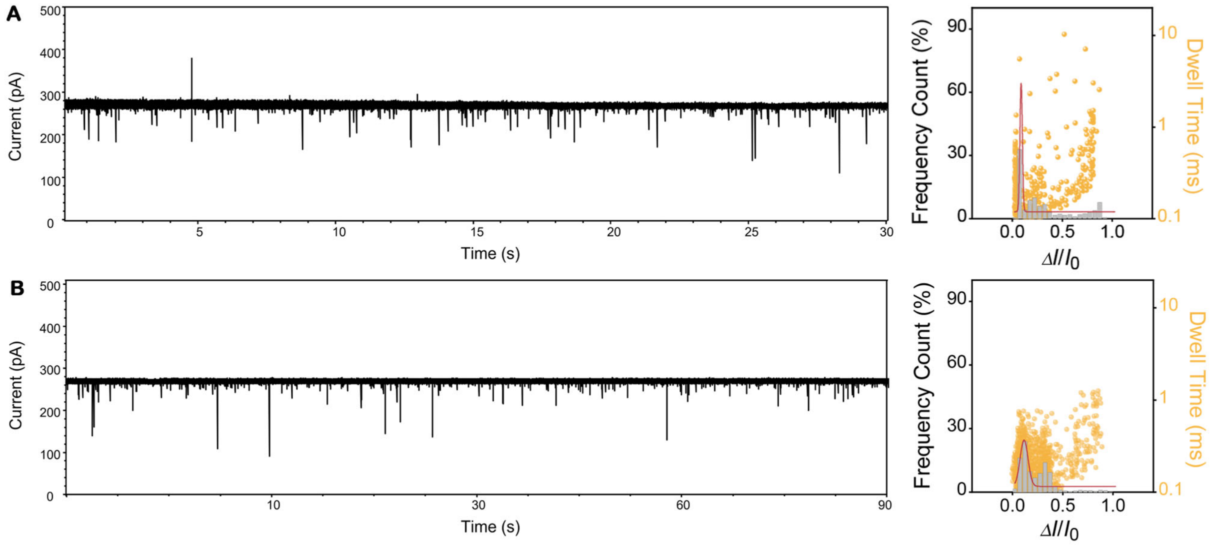

Fig. S4. Nanopore current traces and event analysis for bare PFAS. Representative current traces of 0.001 mM (**A**) PFOA and (**B**) PFOS through α-HL nanopore, together with the corresponding two-dimensional scatter plots of relative blockade versus dwell time of valid events from nanopore results and histograms of normalized event frequency versus current blockade.

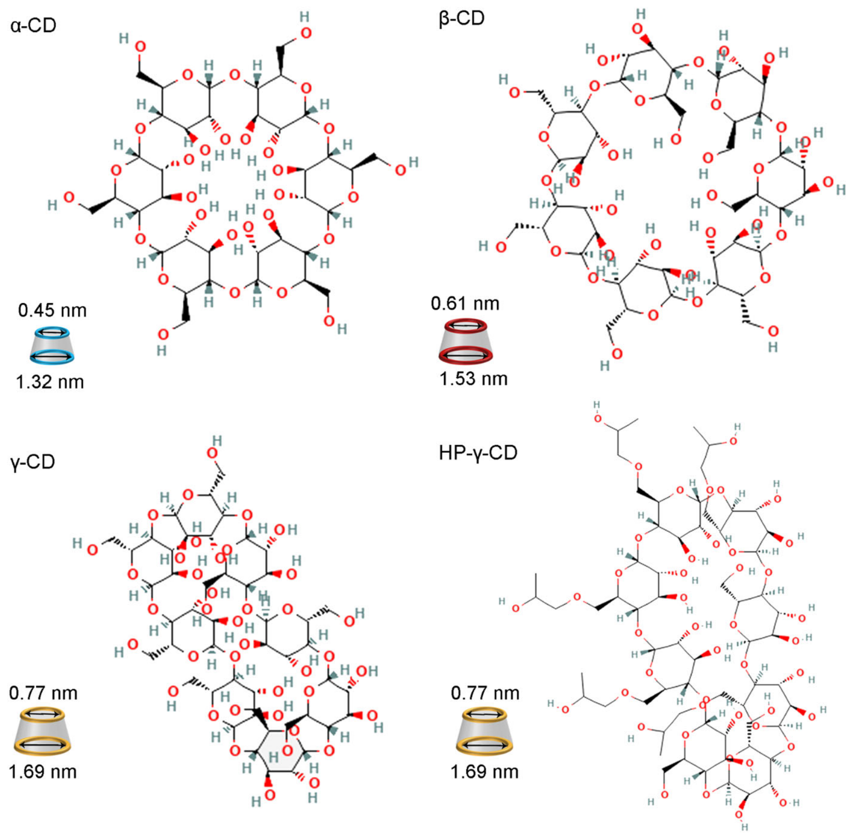

Fig. S5. Chemical structures of α-CD, β-CD, γ-CD, and HP-γ-CD from PubChem. Together with corresponding schematic diagrams and dimensions.

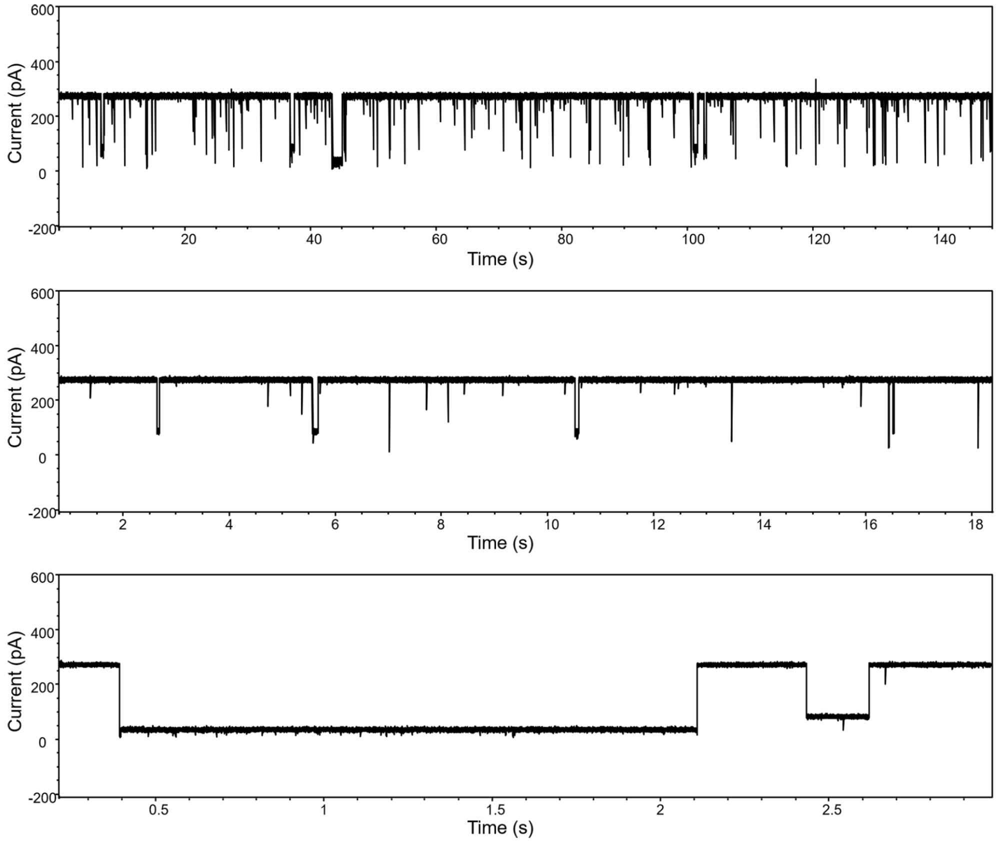

Fig. S6. Representative raw current traces of γ-CD within an α-HL nanopore in different time scales.

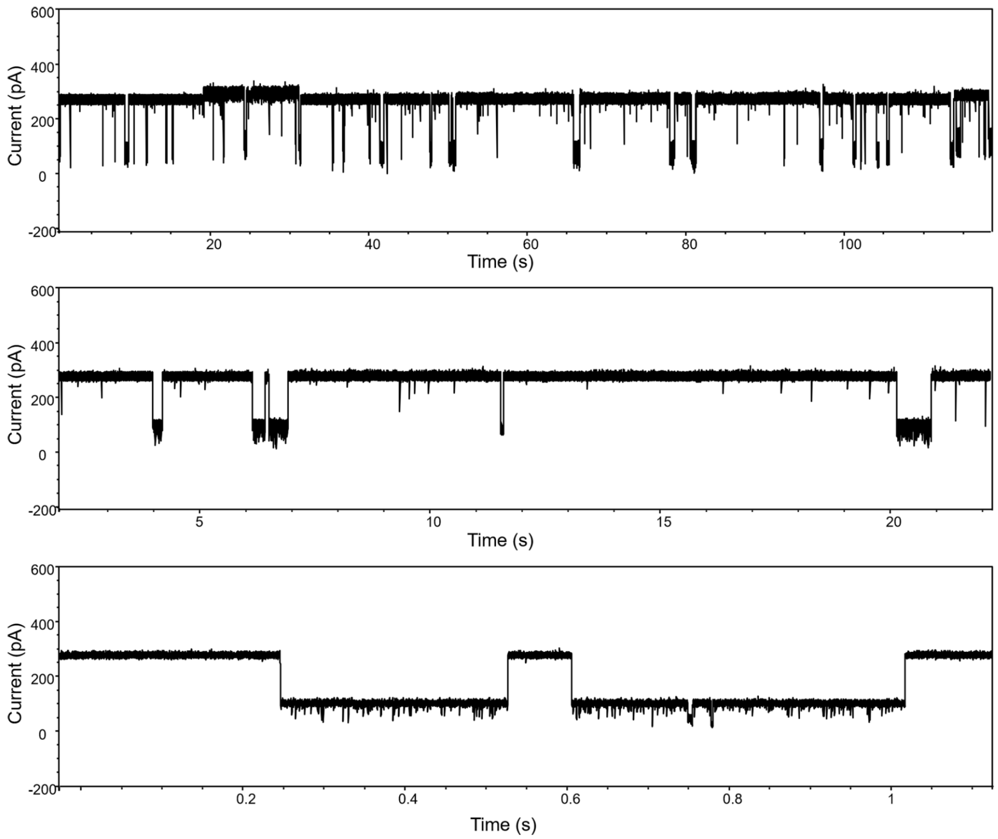

Fig. S7. Representative raw current traces of γ-CD-PFOA within an α-HL nanopore in different time scales.

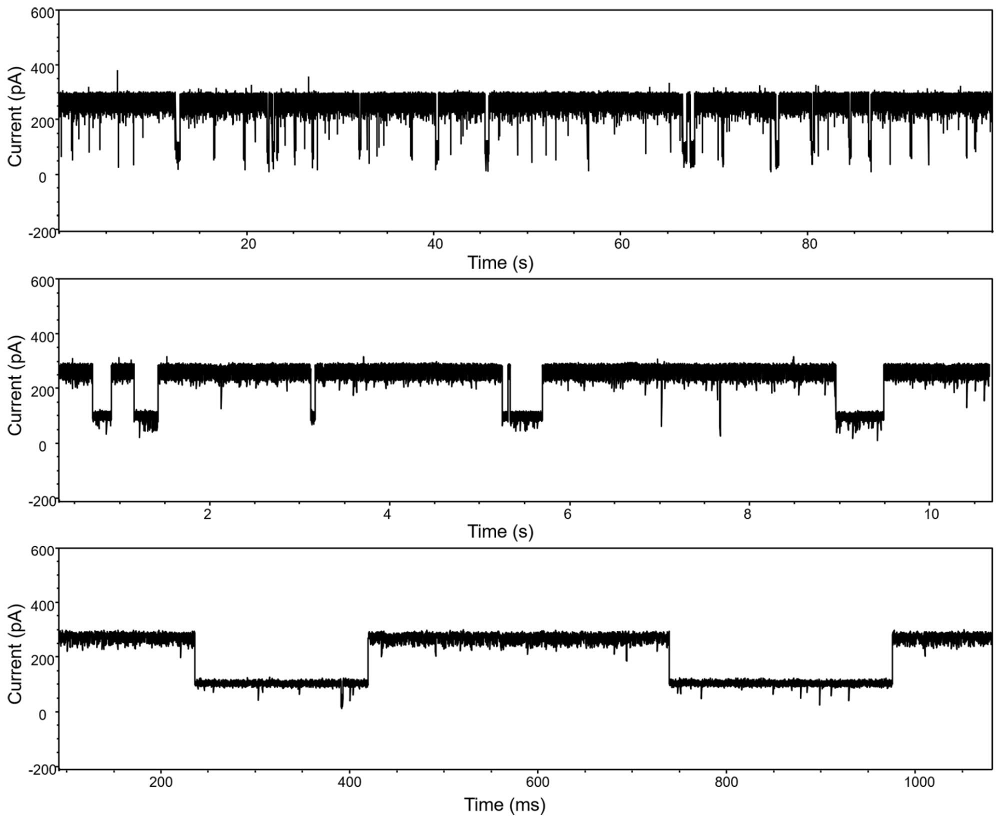

Fig. S8. Representative raw current traces of γ-CD-PFOS within an α-HL nanopore in different time scales.

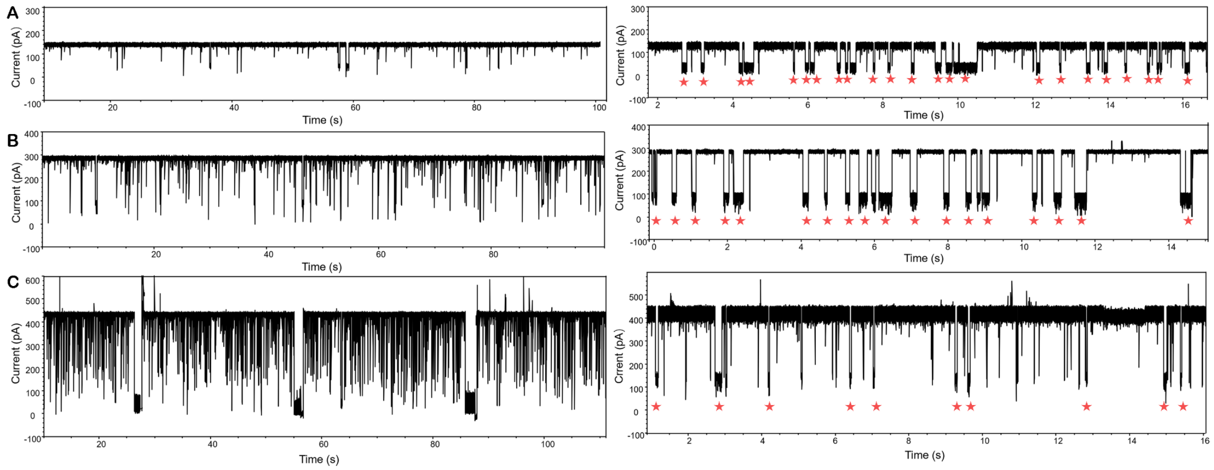

Fig. S9. Representative raw current traces of γ-CD-PFOA within an α-HL nanopore under various transmembrane potentials of (**A**) +50 mV, (**B**) +100 mV, and (**C**) +150 mV. Stars indicate signature signals of γ-CD-PFOA.

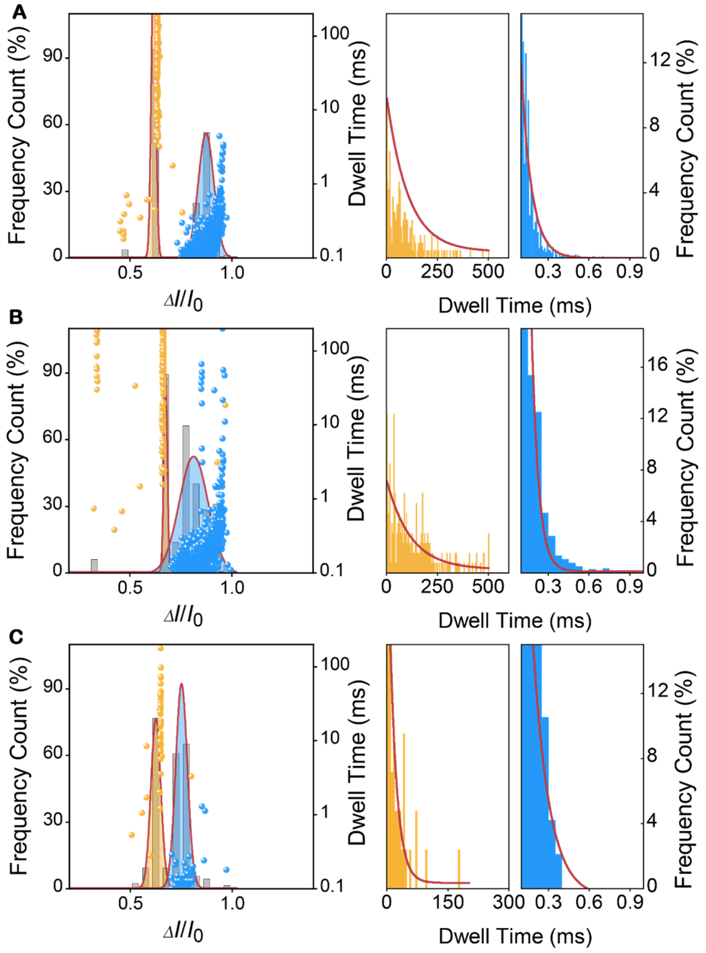

Fig. S10. **Effects of the applied voltage on the interaction between γ-CD and PFOA within nanopore. Left:** two-dimensional scatter plots of relative blockade versus dwell time of valid Level 1 (yellow) and Level 2 (blue) events from nanopore results; **Right:** histograms of normalized event frequency versus current blockade of γ-CD-PFOA within an α-HL nanopore under various transmembrane potentials: (**A**) 50 mV, (**B**) 100 mV, and (**C**) 150 mV.

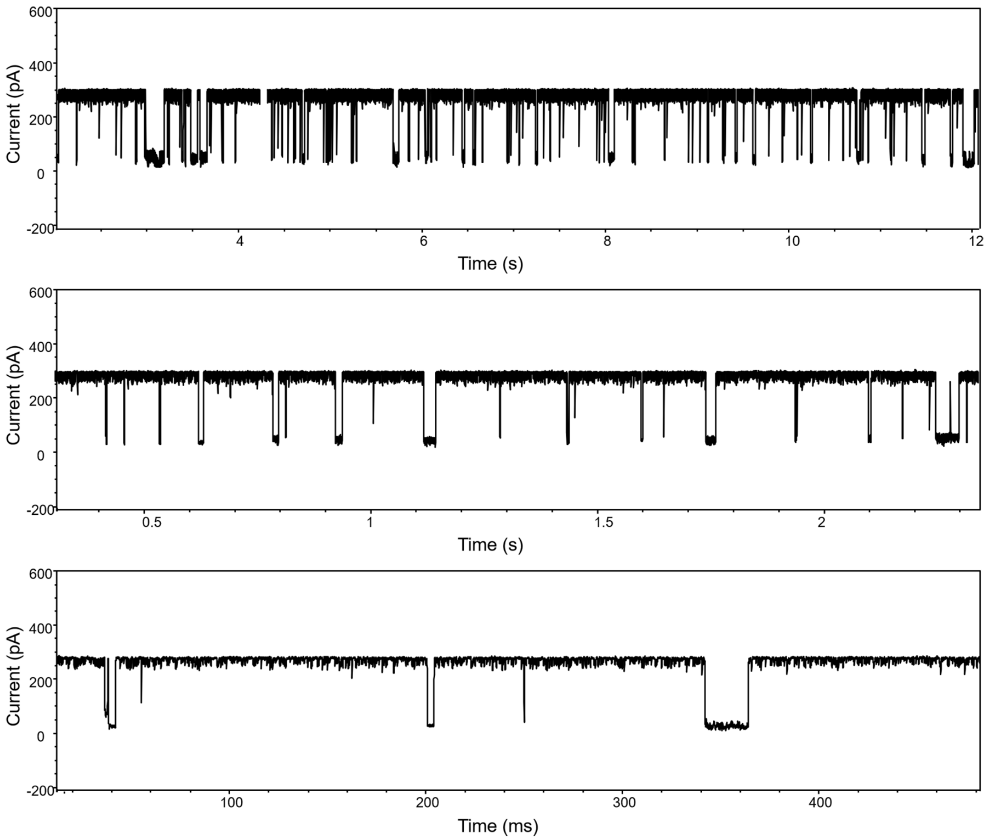

Fig. S11. Representative raw current traces of HP-γ-CD within an α-HL nanopore in different time scales.

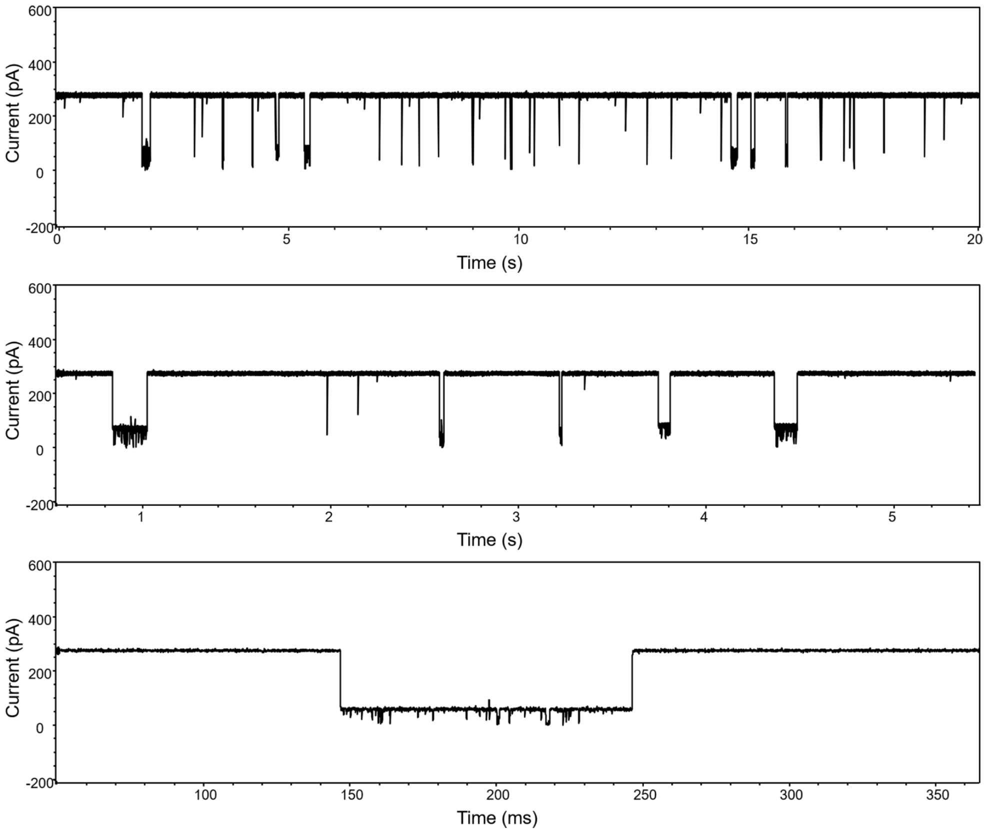

Fig. S12. Representative raw current traces of HP-γ-CD-PFOA within an α-HL nanopore in different time scales.

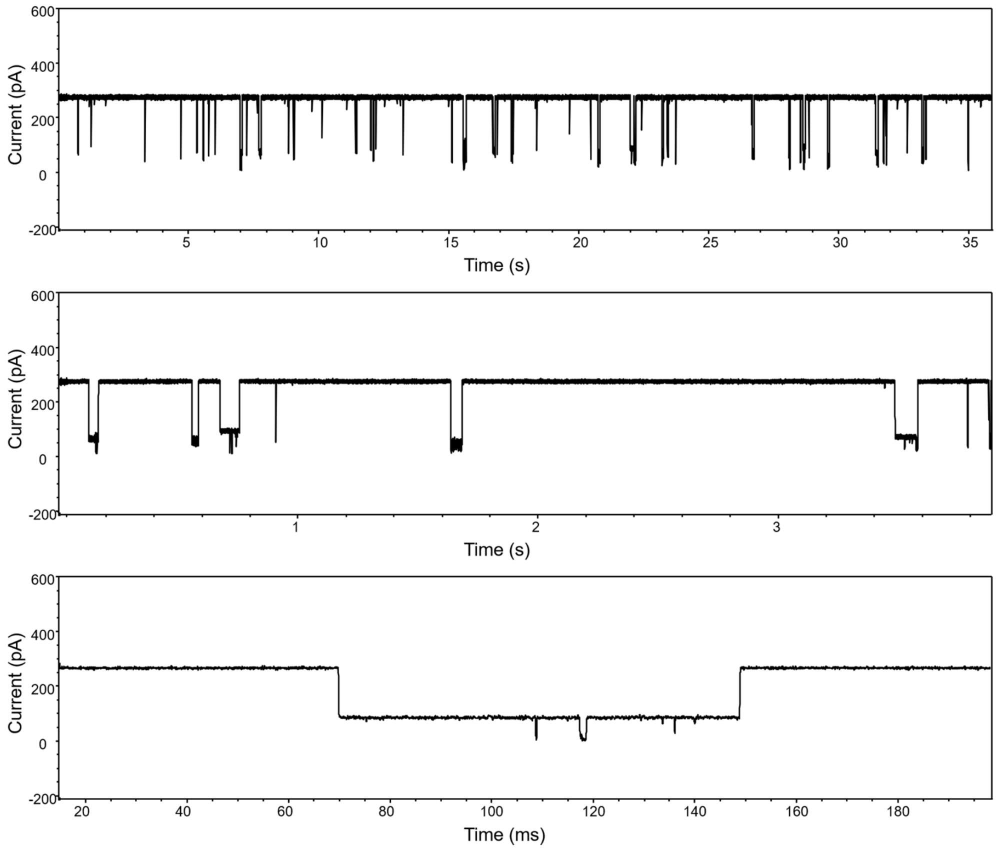

Fig. S13. Representative raw current traces of HP-γ-CD-PFOS within an α-HL nanopore in different time scales.

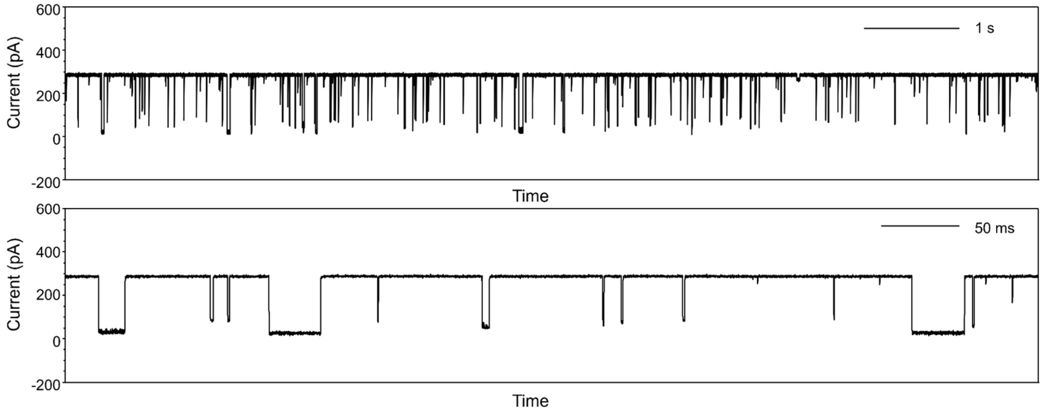

Fig. S14. Representative raw current traces of HP-γ-CD-KCl within an α-HL nanopore in different time scales.

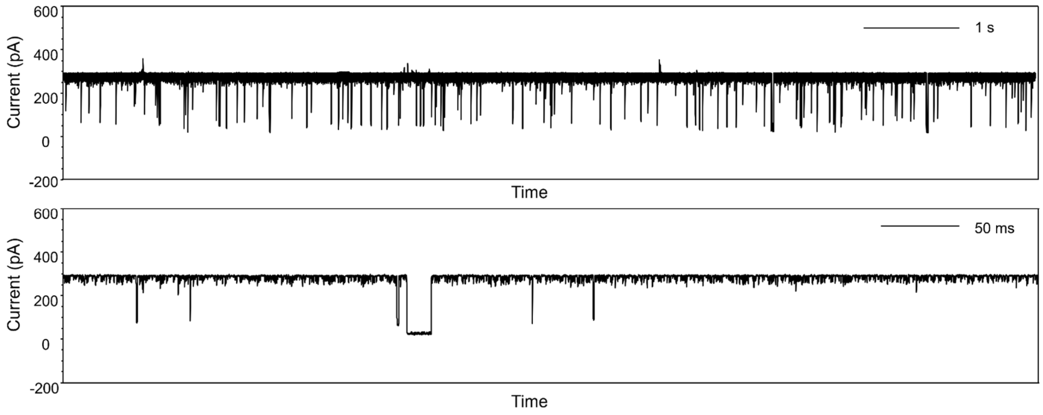

Fig. S15. Representative raw current traces of HP-γ-CD-Na_2SO_4 within an α-HL nanopore in different time scales.

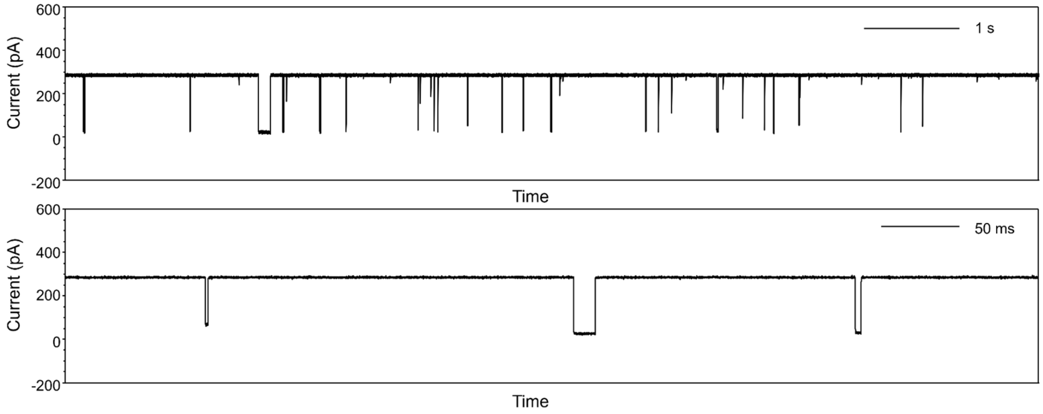

Fig. S16. Representative raw current traces of HP-γ-CD-Na_3PO_4 within an α-HL nanopore in different time scales.

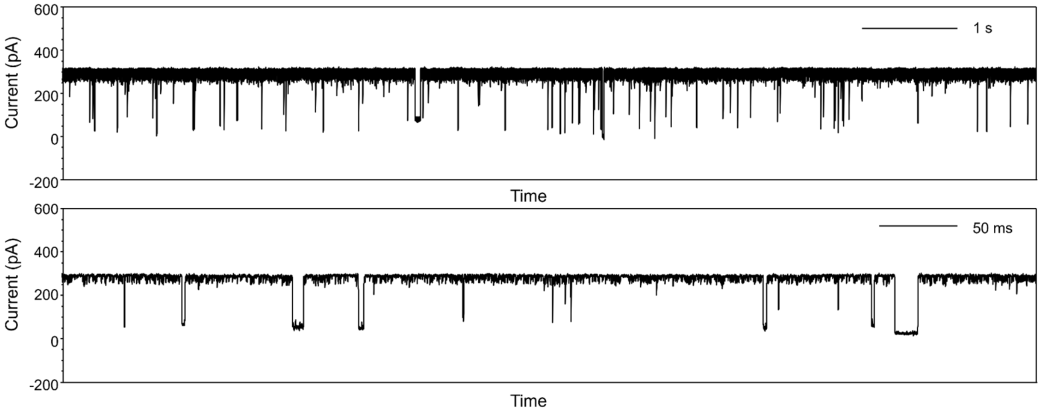

Fig. S17. Representative raw current traces of HP-γ-CD-$C_{18}H_{34}O_2$ within an α-HL nanopore in different time scales.

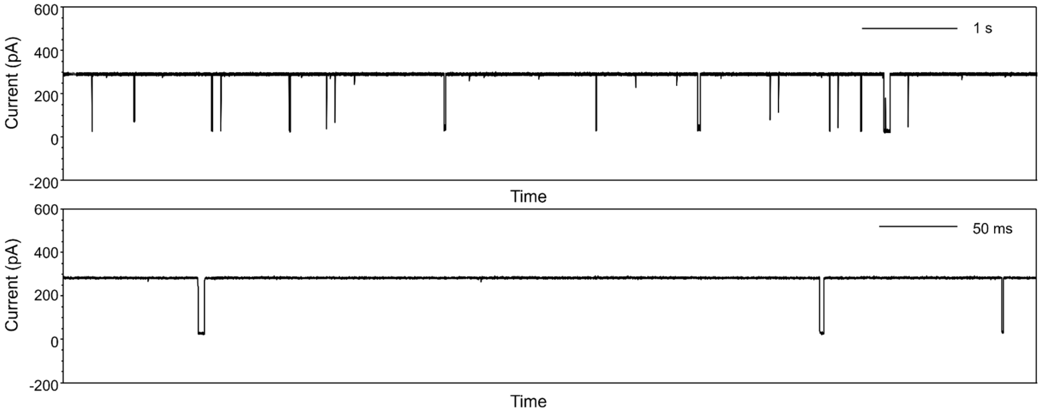

Fig. S18. Representative raw current traces of HP-γ-CD-$C_{18}H_{36}O$ within an α-HL nanopore in different time scales.

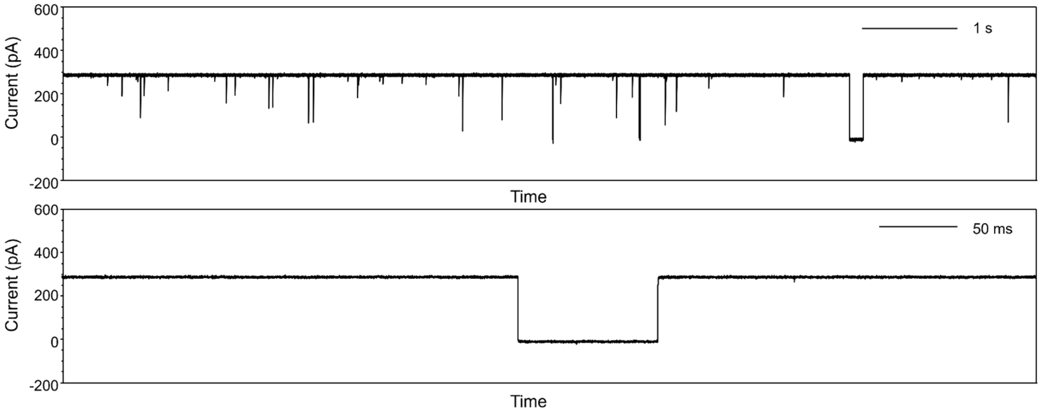

Fig. S19. Representative raw current traces of HP-γ-CD-NaF within an α-HL nanopore in different time scales.

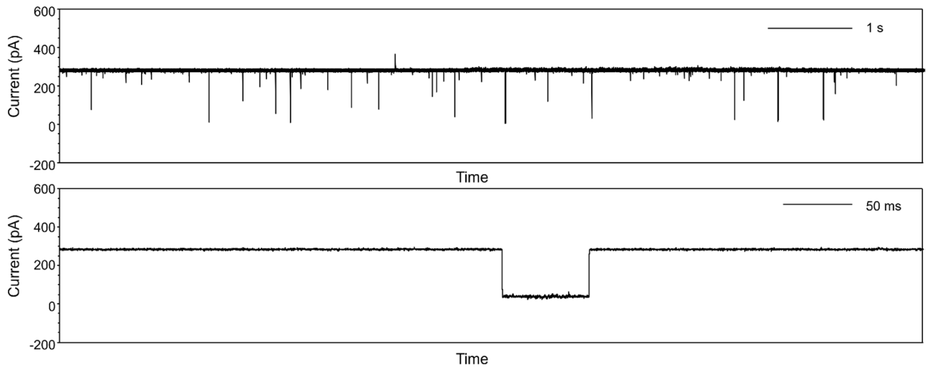

Fig. S20. Representative raw current traces of HP-γ-CD-F_6NaP within an α-HL nanopore in different time scales.

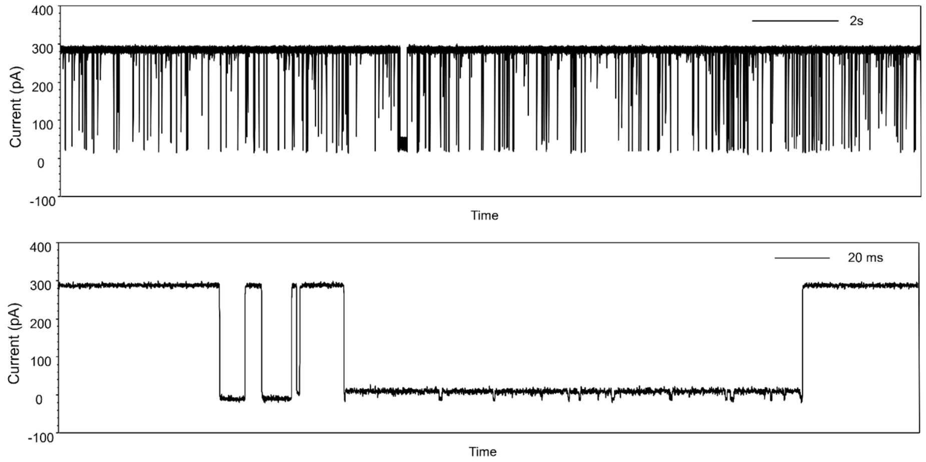

Fig. S21. Representative raw current traces of HP-γ-CD-PFOA within an α-HL nanopore in different time scales. The PFOA concentration is 0.4 ppm.

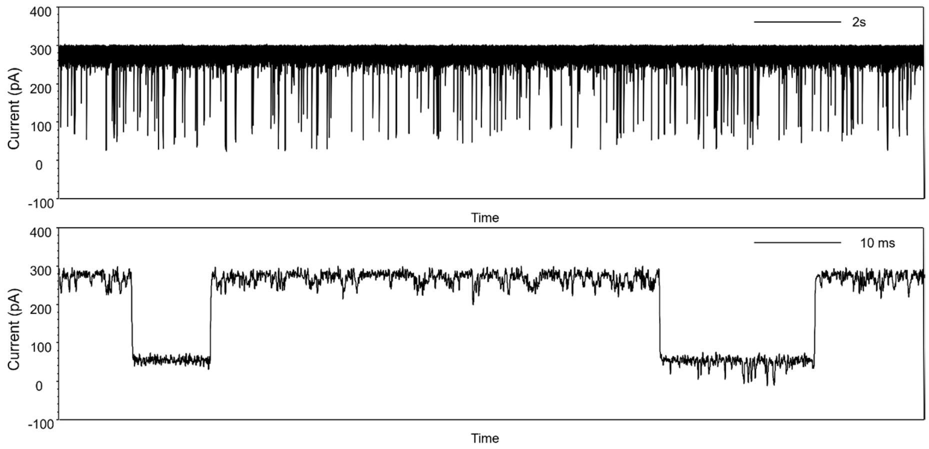

Fig. S22. Representative raw current traces of HP-γ-CD-PFOS within an α-HL nanopore in different time scales. The PFOS concentration is 2 ppm.

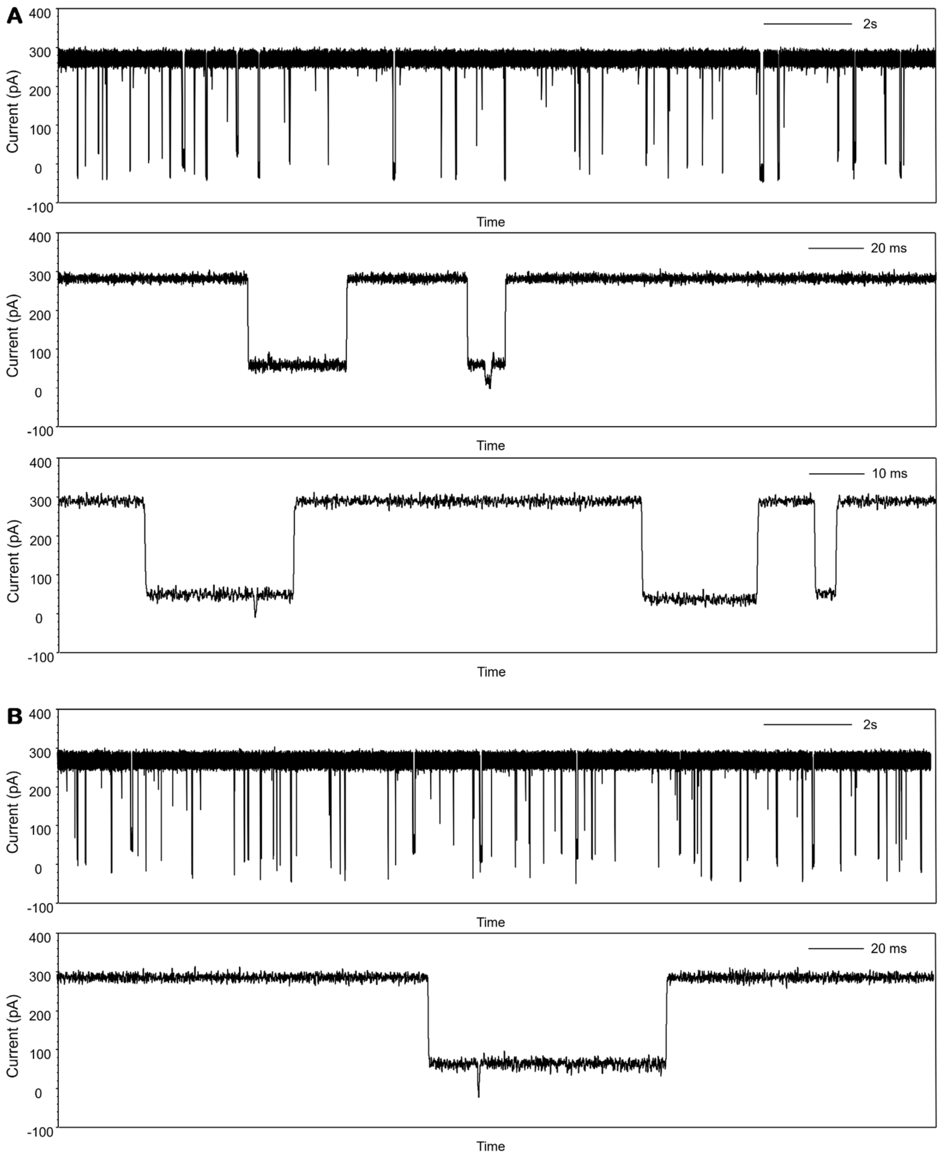

Fig. S23. Representative raw current traces of HP-γ-CD-PFOA within an α-HL nanopore in different time scales. The PFOA concentration is: (**A**) 400 ppt and (**B**) 200 ppt. All samples were tested after ×10000 concentration by rotary evaporation.

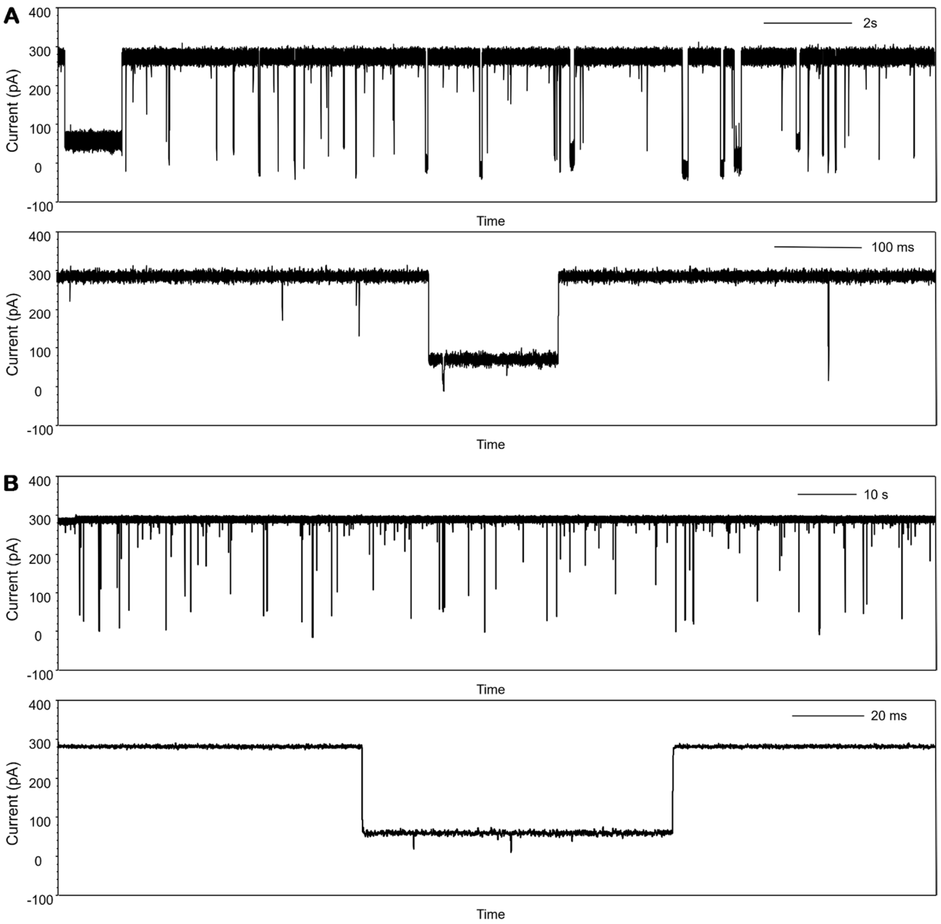

Fig. S24. Representative raw current traces of HP-γ-CD-PFOS within an α-HL nanopore in different time scales. The PFOS concentration is: (**A**) 400 ppt and (**B**) 200 ppt. All samples were tested after ×10000 concentration by rotary evaporation.

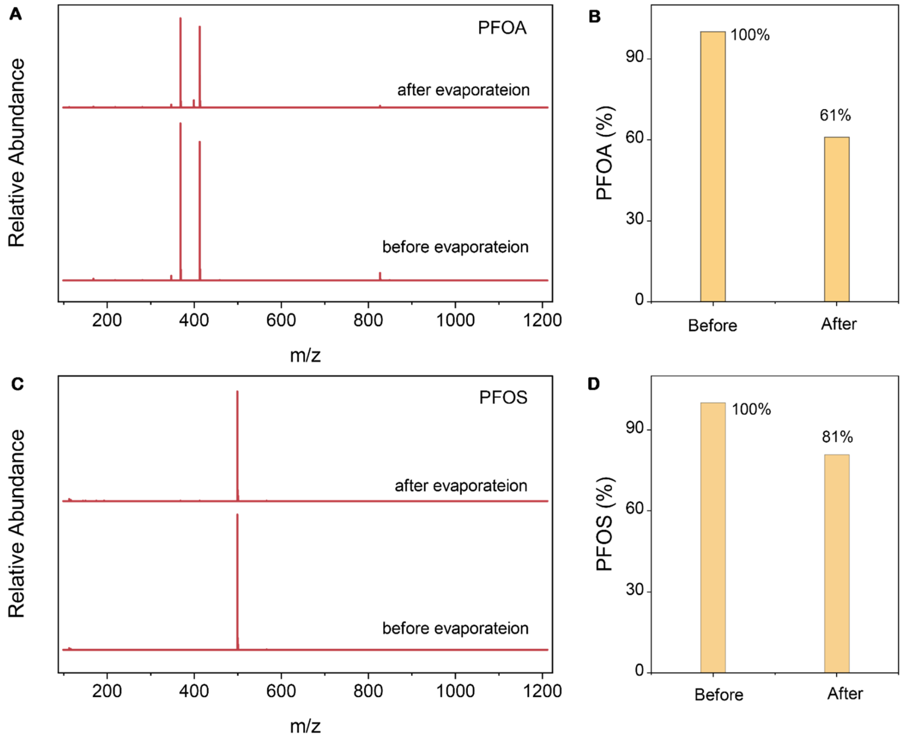

Fig. S25. PFAS mass loss before and after rotary evaporation. **A:** Mass spectrometry characterization of PFOA before and after rotary evaporation. **B:** Estimation of mass loss *via* the abundance of corresponding characteristic peaks. **C:** Mass spectrometry characterization of PFOS before and after rotary evaporation. **D:** Estimation of mass loss *via* the abundance of corresponding characteristic peaks. Note: The initial concentration of the PFOA or PFOS solution was 4 ppm (100 uL) before rotary evaporation. After diluting the solution 10000-fold, rotary evaporation was carried out at 60 °C. Following the reconstitution of the rotary-evaporated sample to 100 uL, a comparative analysis was conducted using mass spectrometry.

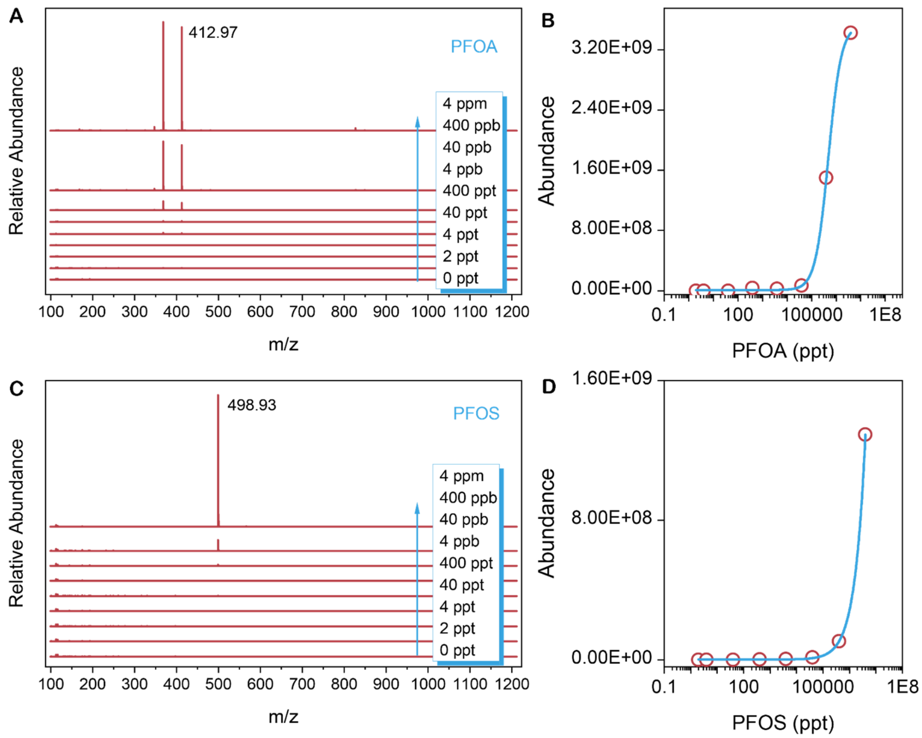

Fig. S26. Sensitivity analysis of PFAS detection by mass spectrometry. A: Mass spectrometry characterizations of PFOA at different concentrations in water within 0-4 ppm range. **B:** Corresponding abundance of the characteristic peak for PFOA (412.97) as a function of PFOA concentration. **C:** Mass spectrometry characterizations of PFOS at different concentrations in water within 0-4 ppm range. **D:** Corresponding abundance of the characteristic peak for PFOS (498.93) as a function of PFOS concentration. The curves in (B) and (D) were fitted by using logistic model to show the trend of abundance values with respect to concentrations.

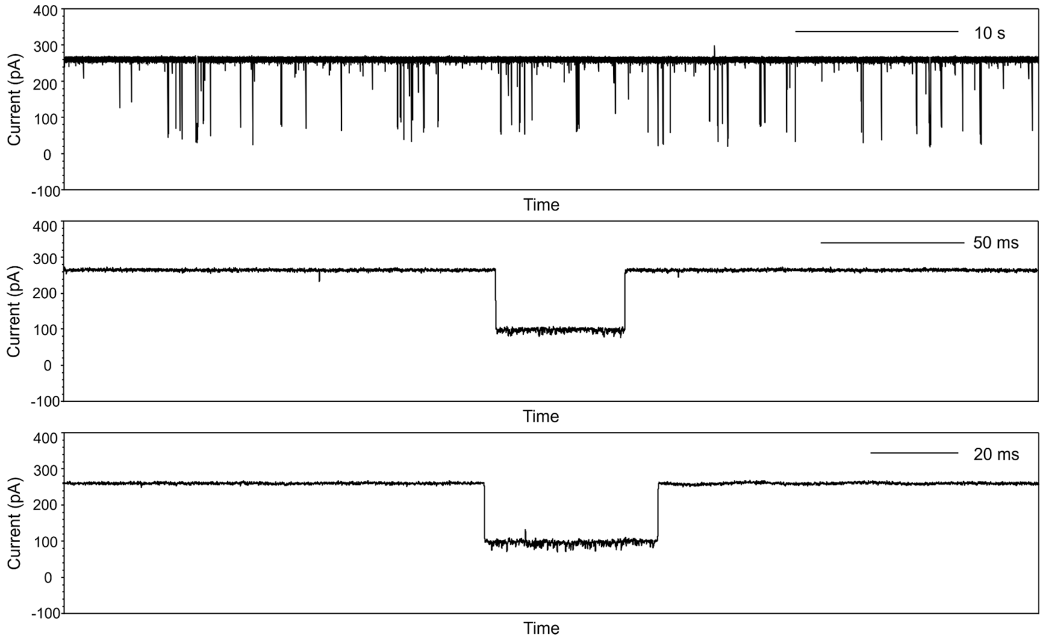

Fig. S27. Representative raw current traces of HP-γ-CD-PFBA (C4) within an α-HL nanopore in different time scales.

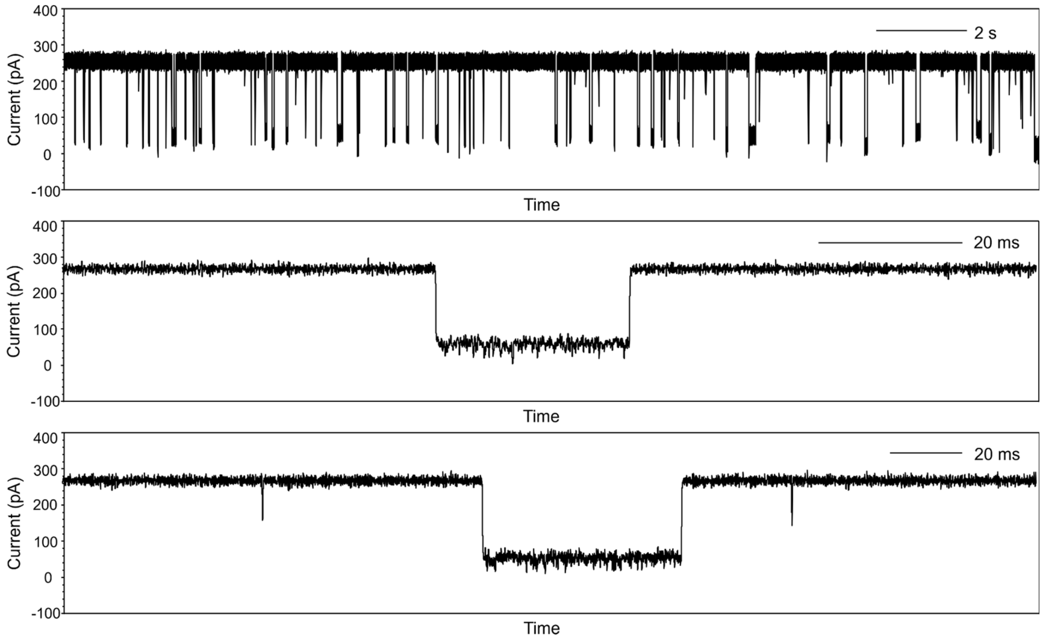

Fig. S28. Representative raw current traces of HP-γ-CD-PFPA (C5) within an α-HL nanopore in different time scales.

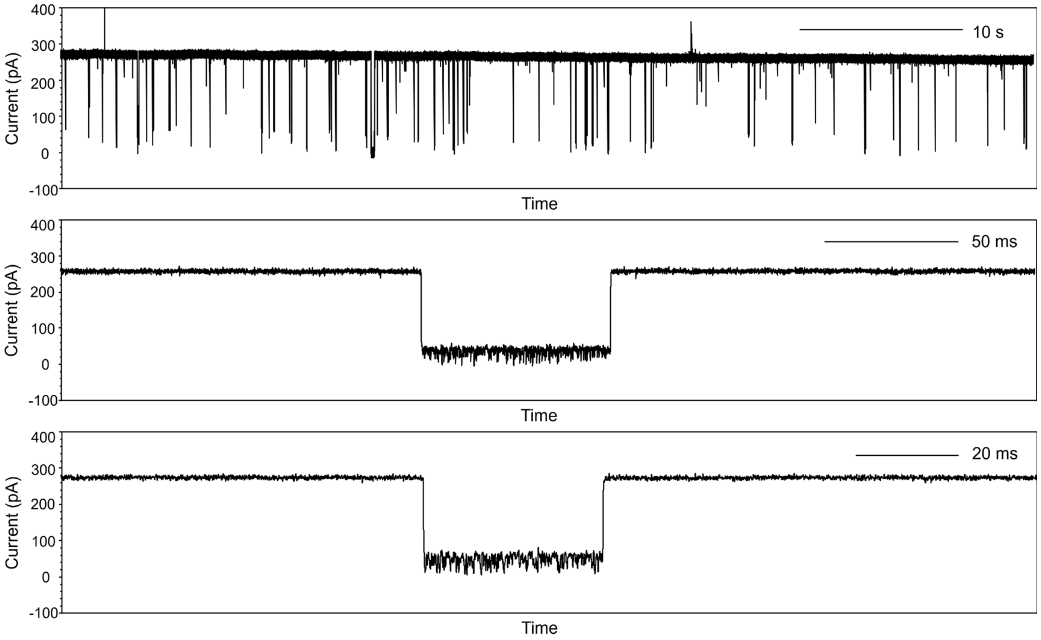

Fig. S29. Representative raw current traces of HP-γ-CD-PFHxA (C6) within an α-HL nanopore in different time scales.

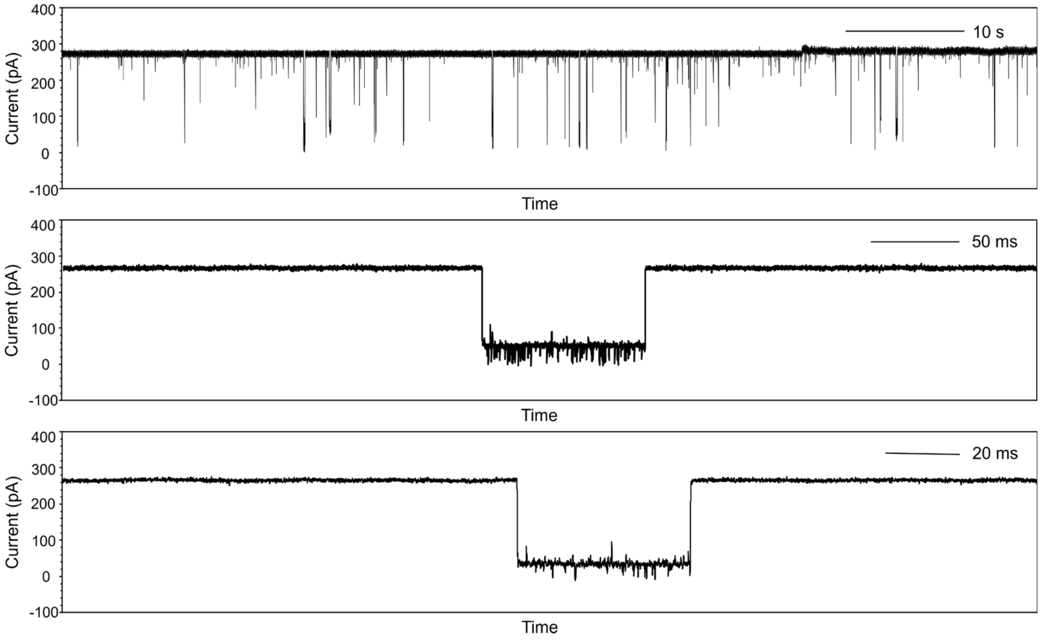

Fig. S30. Representative raw current traces of HP-γ-CD-PFHpA (C7) within an α-HL nanopore in different time scales.

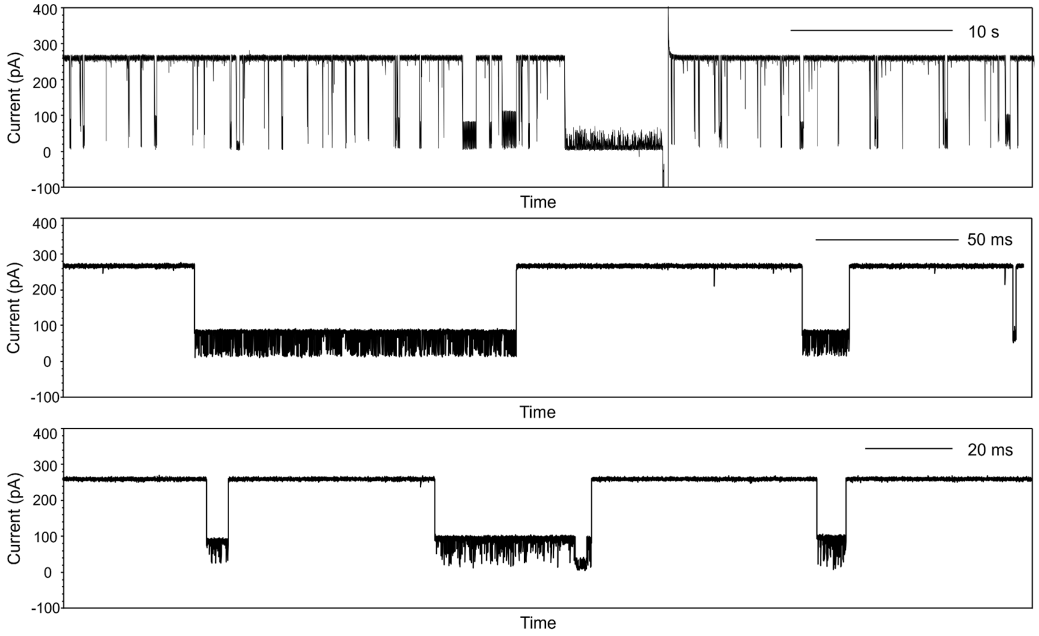

Fig. S31. Representative raw current traces of HP-γ-CD-PFNA (C9) within an α-HL nanopore in different time scales.

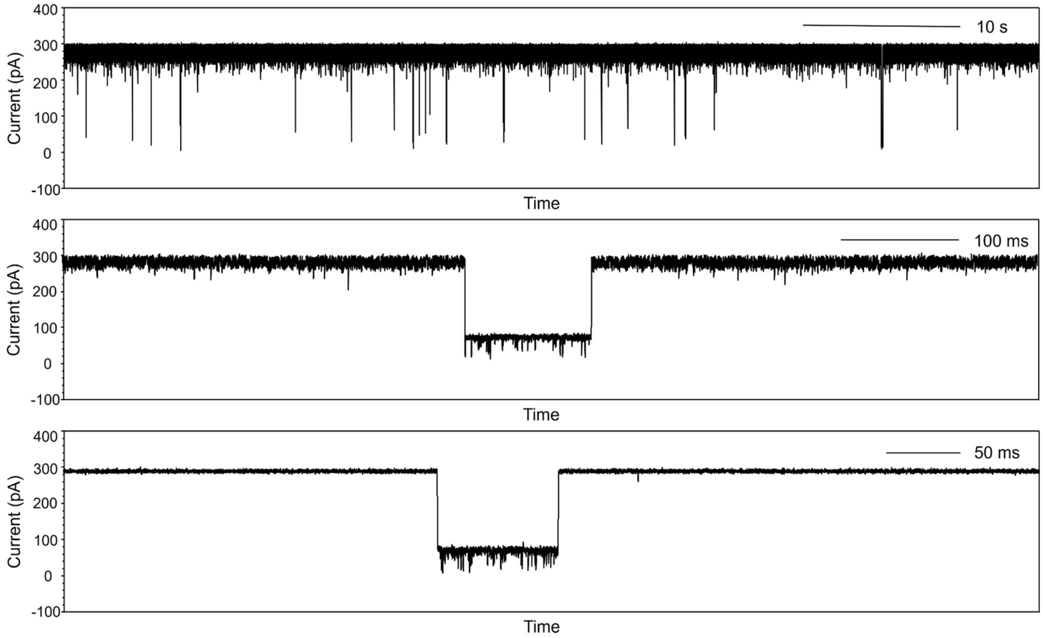

Fig. S32. Representative raw current traces of HP-γ-CD-PFBS(C4) within an α-HL nanopore in different time scales.

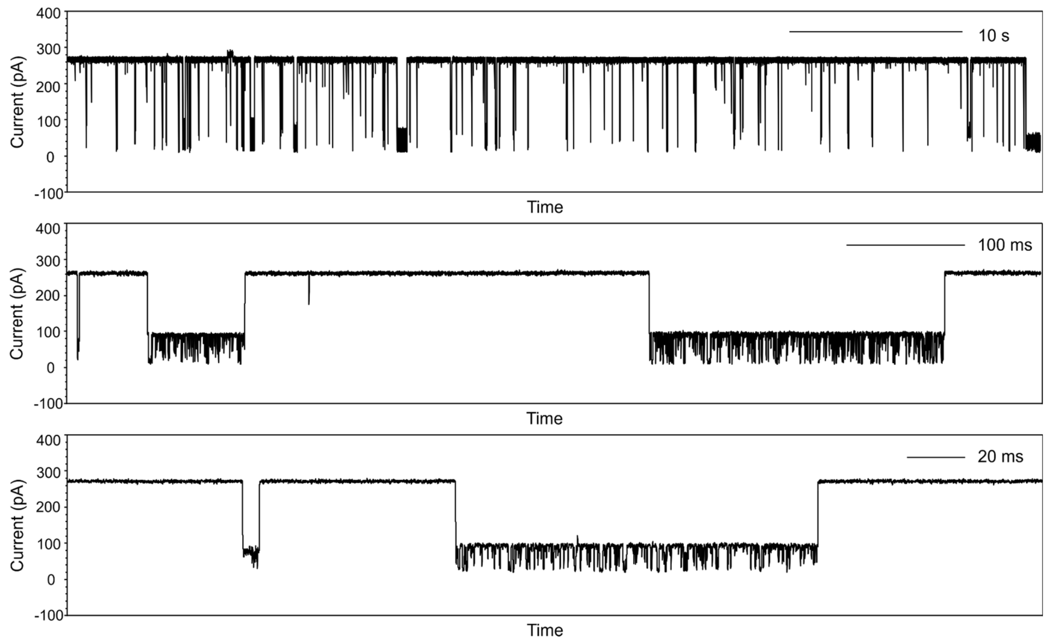

Fig. S33. Representative raw current traces of HP-γ-CD-PFHxS (C6) within an α-HL nanopore in different time scales.

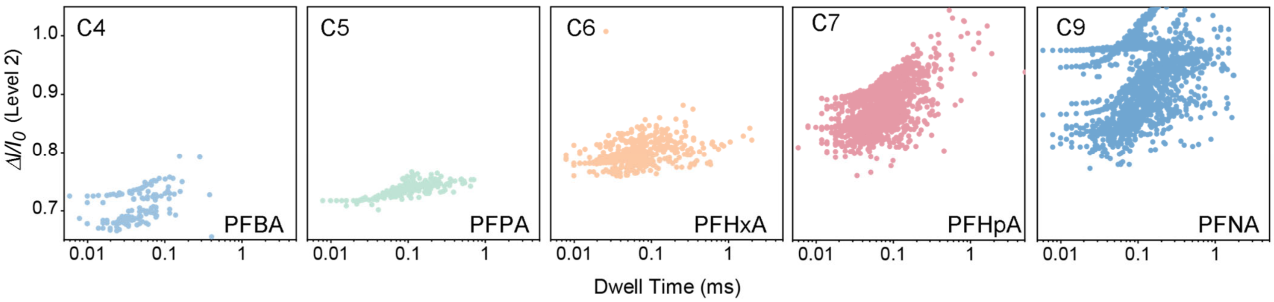

Fig. S34. Two-dimensional scatter plots of relative blockade versus dwell time of valid events from nanopore results of various molecules of the PFSAs family with HP-γ-CD (scatter plots of PFOA shown in Fig. 3F).

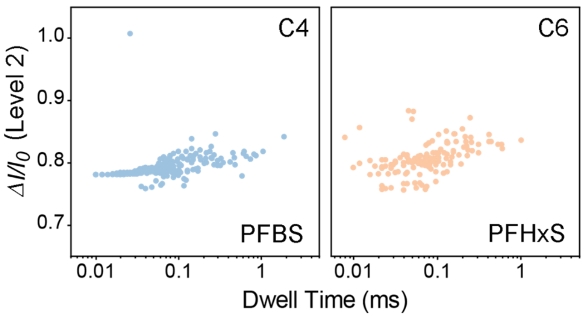

Fig. S35. Two-dimensional scatter plots of relative blockade versus dwell time of valid events from nanopore results of various molecules of the PFCAs family with HP-γ-CD. (scatter plots of PFOS shown in Fig. 3H).

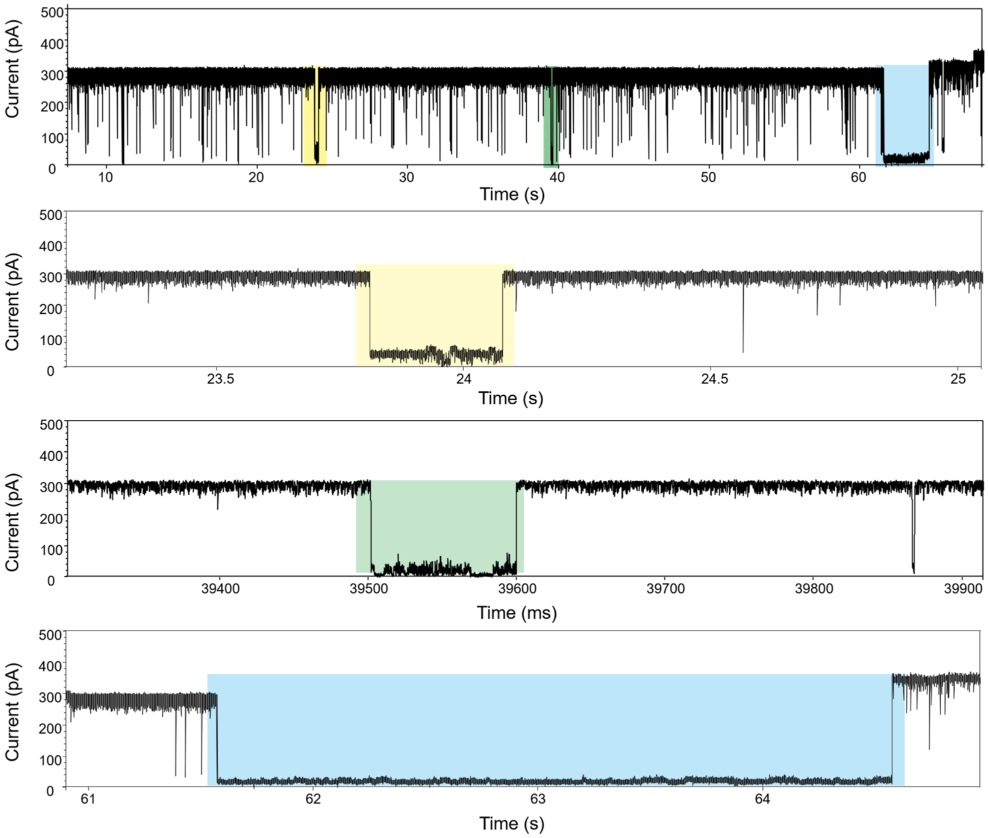

Fig. S36. Representative raw current traces of HP-γ-CD-PFDA (C10) within an α-HL nanopore in different time scales.

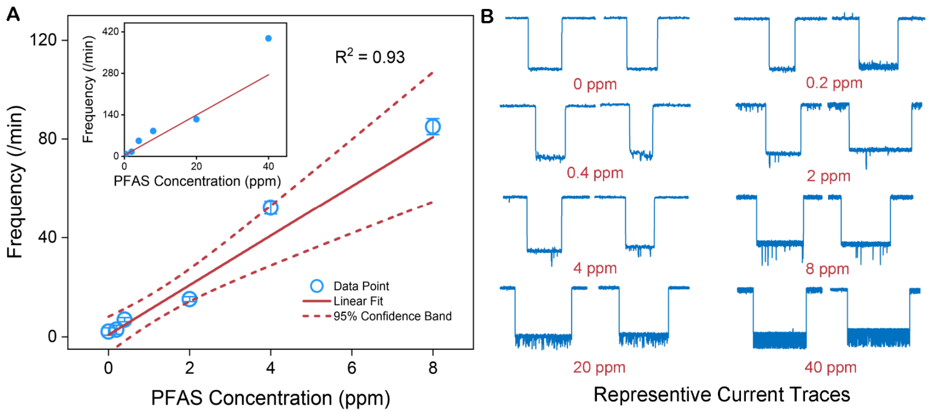

Fig. S37. Sensitivity of HP-γ-CD mediated nanopore detection of PFAS mixture. A: Correlation between host-guest interaction induced Level 2 signal frequency and the concentration of PFAS mixtures within 0-8 ppm range in water. Inset shows correlation within 0-40 ppm range. Data represents mean ± SD of replicates (n=3 statistically independent experiments). **B:** Representative raw current traces of HP-γ-CD-PFAS mixture with different concentrations within an α-HL nanopore.

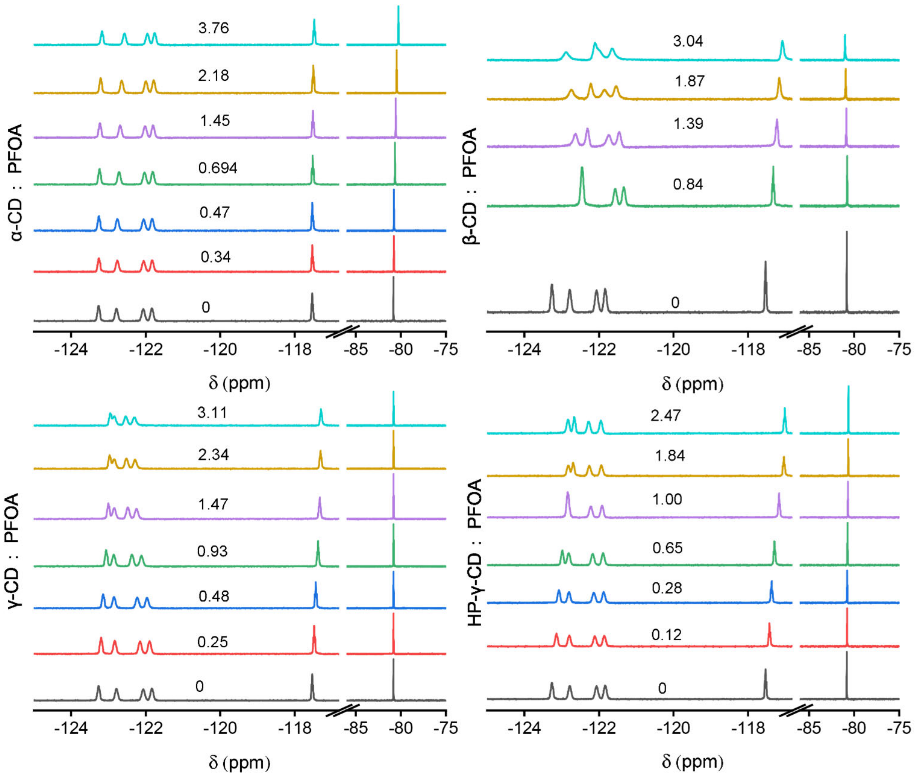

Fig. S38. ^{19}F NMR spectra of α-CD, β-CD, γ-CD, and HP-γ-CD with PFOA at various molar ratios in D_2O.

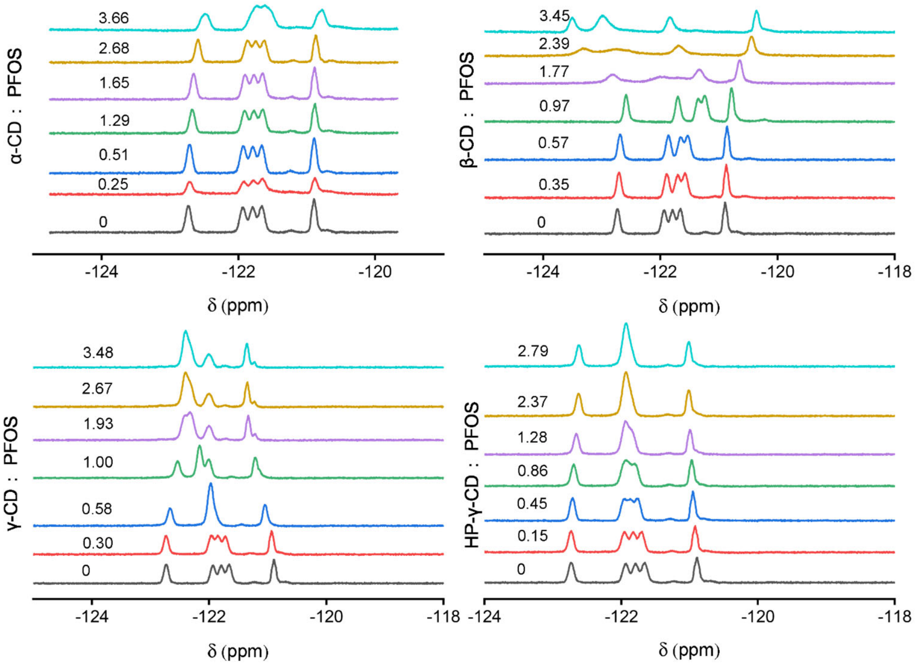

Fig. S39. ^{19}F NMR spectra of α-CD, β-CD, γ-CD, and HP-γ-CD with PFOS at various molar ratios in D_2O.

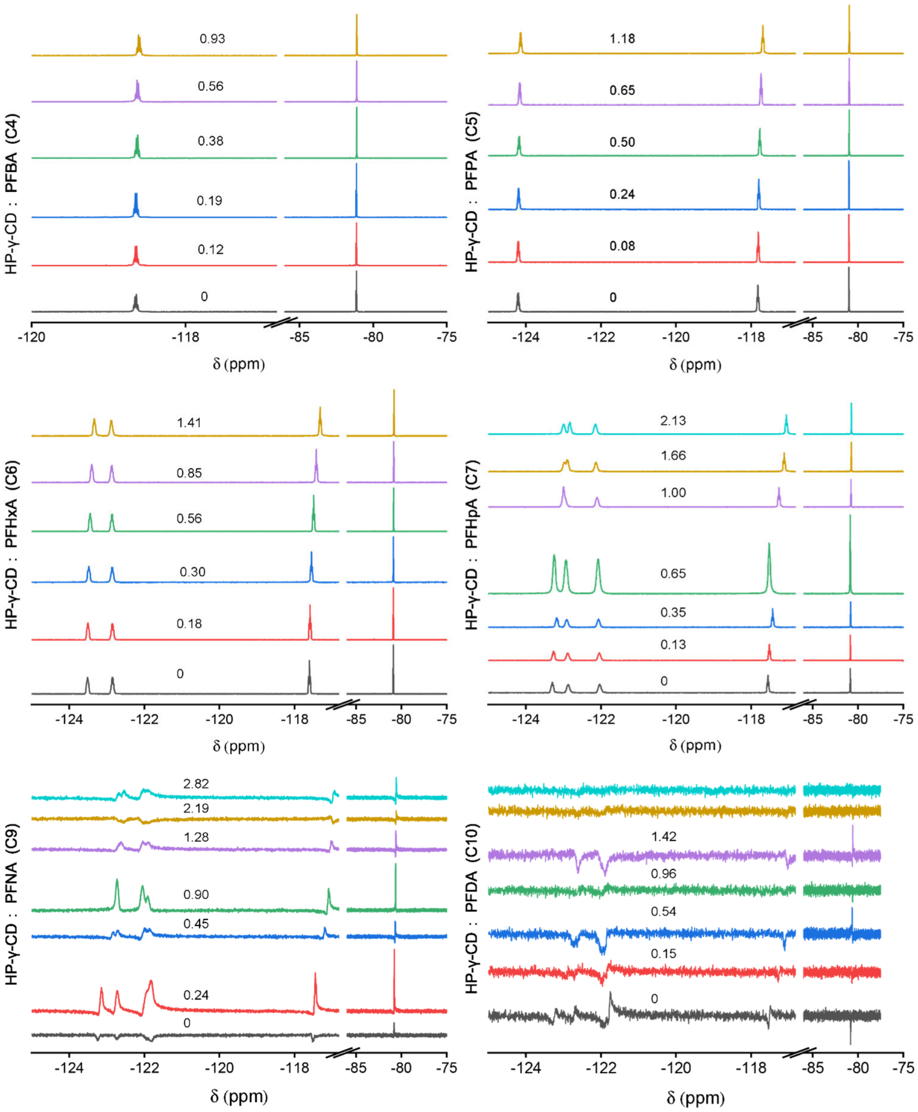

Fig. S40. ^{19}F NMR spectra of HP-γ-CD with molecules of the PFCAs family at various molar ratios in D_2O.

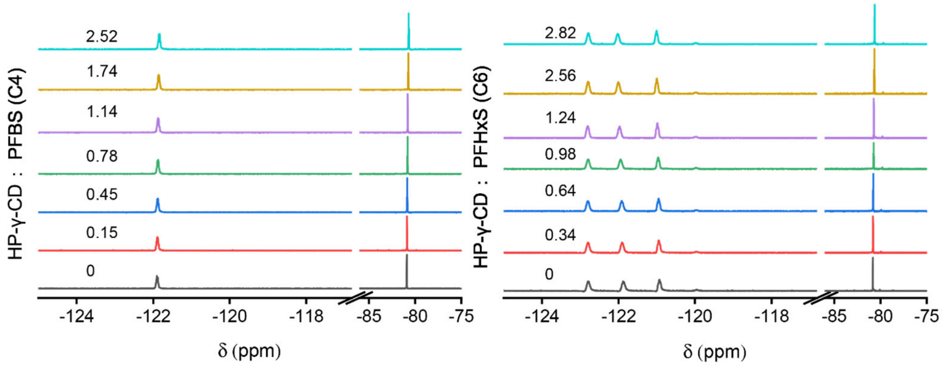

Fig. S41. ^{19}F NMR spectra of HP-γ-CD with molecules of the PFSAs family at various molar ratios in D_2O.

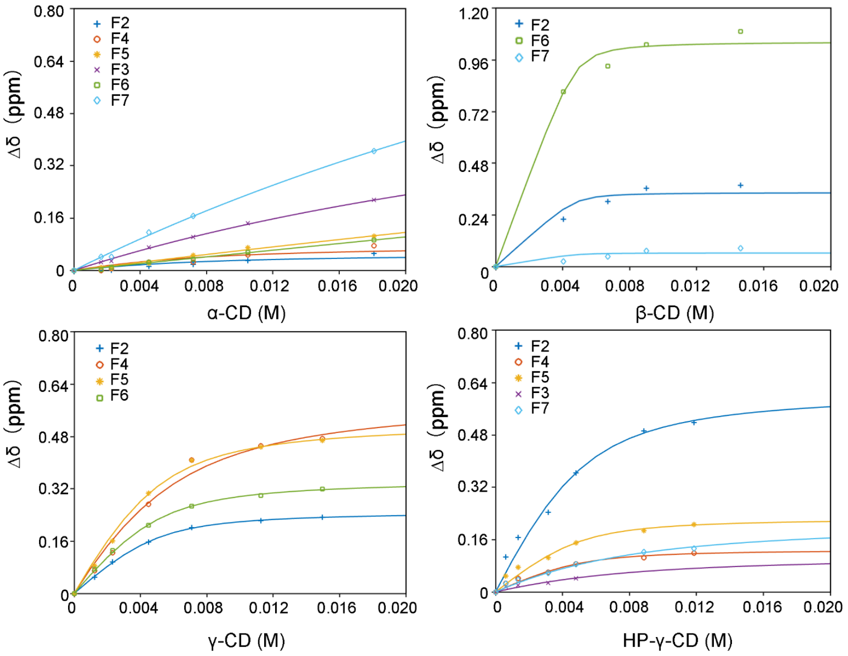

Fig. S42. Plots of change in observed chemical shift of each fluorine of PFOA as a function of total CD concentration of various CD molecules, fitted with the non-linear least-squares regression analysis to determine the association constants.

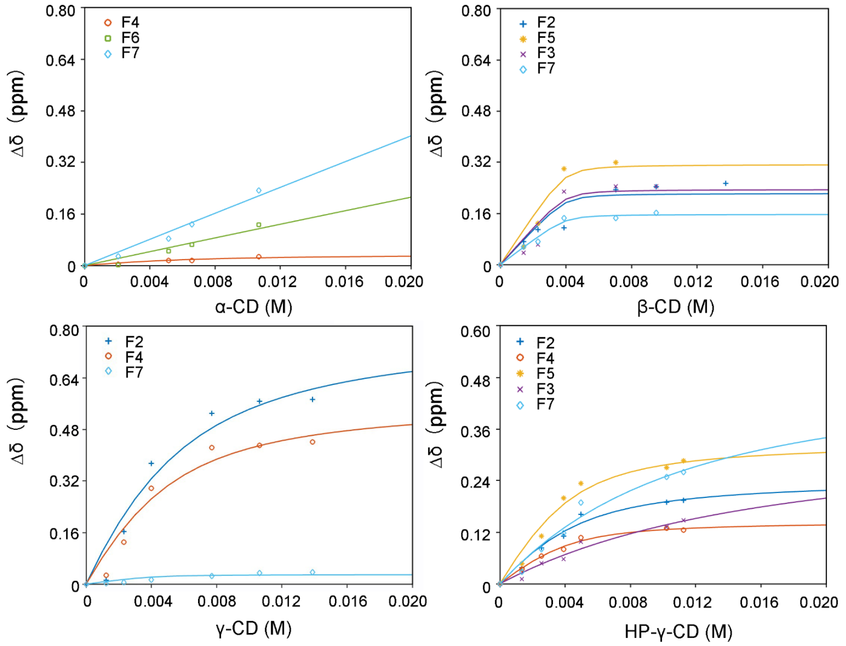

Fig. S43. Plots of change in observed chemical shift of each fluorine of PFOS as a function of total CD concentration of various CD molecules, fitted with the non-linear least-squares regression analysis to determine the association constants.

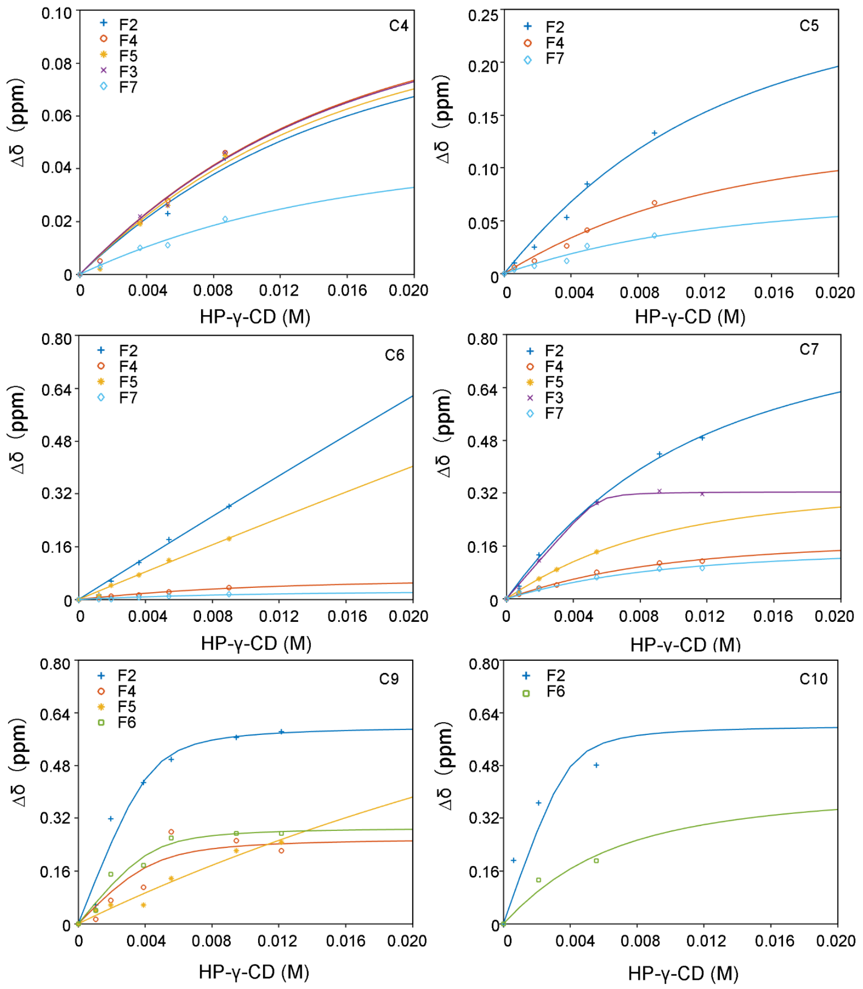

Fig. S44. Plots of change in observed chemical shift of each fluorine for each member of the PFCAs family with carbon chain lengths from C4 to C9 as a function of total HP-γ-CD concentration, fitted with the non-linear least-squares regression analysis to determine the association constants.

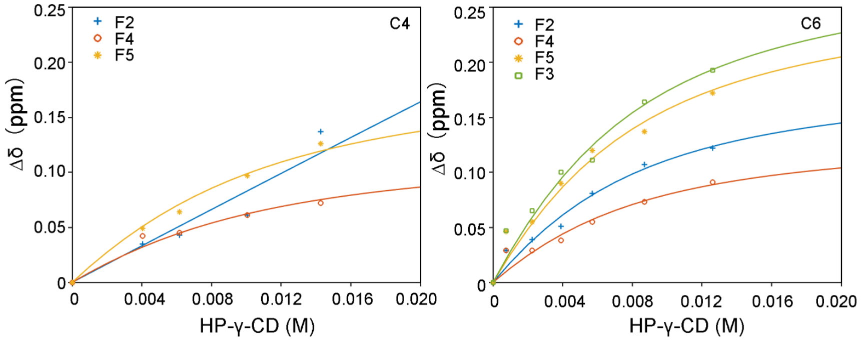

Fig. S45. Plots of change in observed chemical shift of each fluorine for each member of the PFSAs family with carbon chain lengths of C4 and C6 as a function of total HP-γ-CD concentration, fitted with the non-linear least-squares regression analysis to determine the association constants.

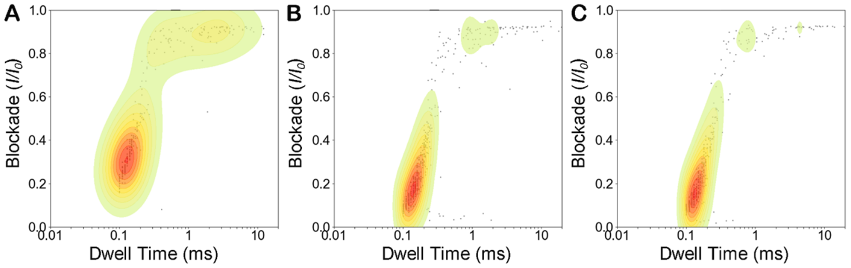

Fig. S46. Two-dimensional contour plots of current blockade versus dwell time of valid events from nanopore results of (**A**) α-CD, (**B**) α-CD-PFOA, and (**C**) α-CD-PFOS.

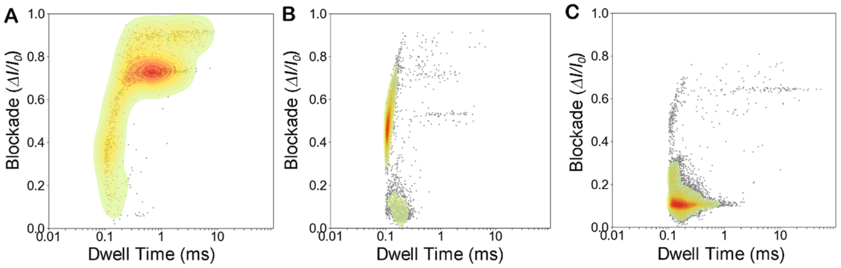

Fig. S47. Two-dimensional contour plots of current blockade versus dwell time of valid events from nanopore results of (**A**) β-CD, (**B**) β-CD-PFOA, (**C**) β-CD-PFOS.

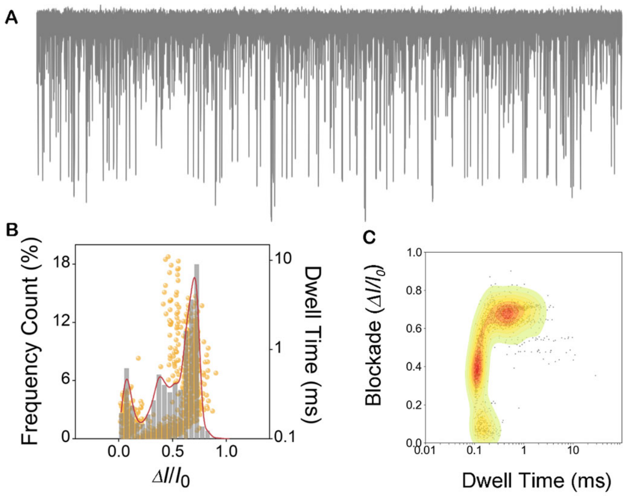

Fig. S48. Analyzing a mixture of β-CD, PFOA, and PFOS using an α-HL nanopore. A: Raw current traces of the mixture. **B:** Corresponding two-dimensional scatter plots of relative blockade versus dwell time of valid events from nanopore results and histograms of normalized event frequency versus current blockade. **C:** Two-dimensional contour plot of current blockade versus dwell time of valid events from nanopore results.

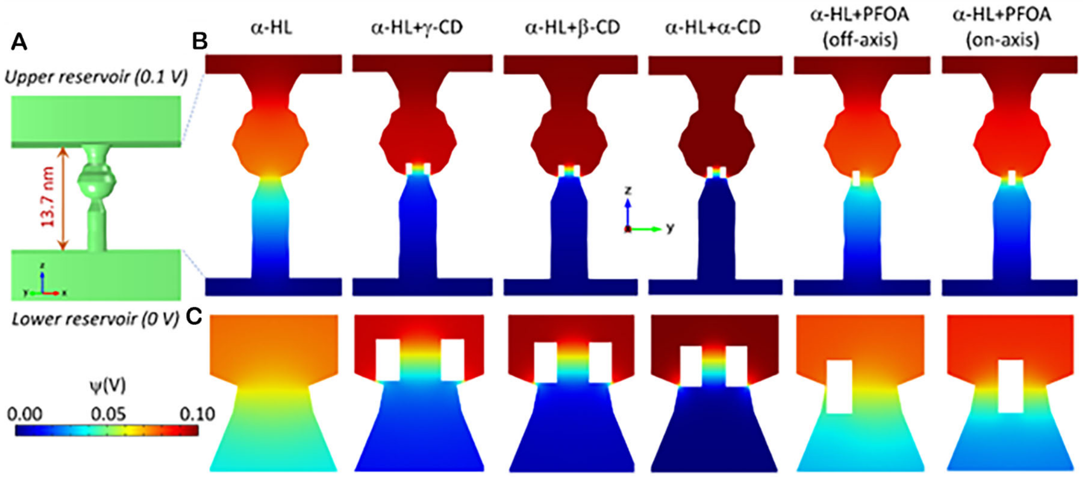

Fig. S49. Ion transport modeling of α-HL, CDs, and PFAS molecules. A: Scheme showing the cavity of α-HL connecting the upper and the lower reservoirs. The region in green is accessible to the ions. A potential bias of 0.1 V is applied between these two reservoirs. **B:** Colormaps of the electrostatic potential along a cut of the pore for α-HL and α-HL hosting different molecules in this study (the region occupied by the molecule is shown in white). **C:** Enlargement of the region near the constriction for the panels shown in B.

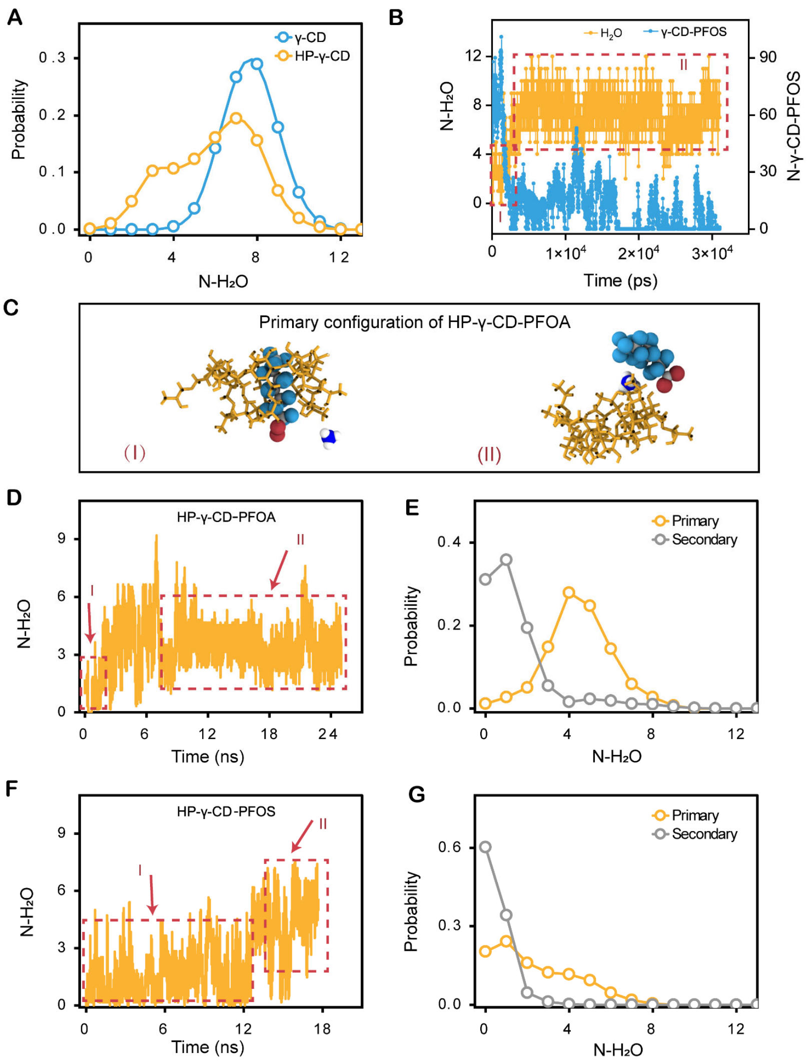

Fig. S50. Molecular simulation of the interaction between cyclodextrins and PFOA/PFOS molecules. A: Probability distribution of H_2O molecules inside the 4.5Å sphere of γ-CD and HP-γ-CD in pure water. **B:** Time evolution of instantaneous number of H_2O molecules in the 4.5Å sphere inside γ-CD inner cavity and number of contacts between PFOS and γ-CD atoms (within 4.0 Å distance). **C-G:** Interaction simulation of (**C-E**) HP-γ-CD-PFOA and (**F-G**) HP-γ-CD-PFOS. **C:** Corresponding snapshots of HP-γ-CD-PFOA with "primary" configuration at the beginning (I) and the end (II) of the trajectory. **D:** Time evolution of instantaneous number of H_2O molecules inside HP-γ-CD-PFOA with "primary" configuration at the beginning (I) and end (II) of the trajectory. The red dashed box and arrows indicate the corresponding configuration. **e:** Probability distribution of H_2O molecules for both "primary" and "secondary" configurations of HP-γ-CD-PFOA system. **F:** Time evolution of instantaneous number of H_2O molecules inside HP-γ-CD-PFOS with "primary" configuration at the beginning (I) and end (II) of the trajectory. The red dashed box and arrows indicate the corresponding configuration (snapshots given in the main manuscript). **G:** Probability distribution of H_2O molecules for both "primary" and "secondary" configurations of HP-γ-CD-PFOS system.

Table S1. The mean relative blockade ($\Delta I/I_0$) and dwell time (τ) of Level 1 and Level 2 signals for γ-CD or HP-γ-CD with PFOA or PFOS.

		Level1 $\Delta I/I_0$	Level 2 $\Delta I/I_0$	Level 1 Dwell Time (ms)	Level 2 Dwell (ms)
γ-CD	PFOA	0.661±0.023	0.781±0.042	16.7±2.001	0.055±0.002
	PFOS	0.676±0.043	0.799±0.121	2.631±0.415	0.038±0.001
	Blank	0.674±0.048	-	0.185±0.002	-
HP-γ-CD	PFOA	0.712±0.091	0.898±0.124	16.9±1.230	0.087±0.001
	PFOS	0.667±0.114	0.806±0.079	29.27±3.563	0.0878±0.001
	Blank	0.863±0.037	-	0.313±0.018	-
γ-CD-PFOA	50 mV	0.615±0.022	0.873±0.076	104.475±20.460	0.076±0.003
	100 mV	0.675±0.017	0.812±0.142	117.516±25.734	0.0503±0.002
	150 mV	0.625±0.048	0.751±0.057	17.0198±2.331	0.119±0.034

Table S2. Examples of different sensing techniques for PFAS analysis.

Analytes	Sensing methods	Detection Techniques	Assay Mechanism	LOD (ppb)	Concentration Range	Cost	Portability	Fabrication complexity	ref
PFAS	Ionization and Mass measurement	HPLC-MS	Solid-phase extraction (SPE)	0.71–16 ppt		High	Low	Complicate	55,56
PFOA/PFOS	Indicator displacement	Spectrophotometer	Guanidinocalix[5]arenes	PFOS:.10.3 ppb<br>PFOA: 10.9 ppb	PFOS:0-3001 ppb<br>PFOA:0-2484 ppb	Low	Good	Moderate	1
PFAS	Fluorescence	Spectrophotometer	imprint-and-report-sensing	5–40 nM		Low	Good	Moderate	57
PFOS	Fluorescence	Spectrophotometer	Nitrogen doped Carbon dots	13.9 ppb	0-1 ppm	Low	High	Moderate	58
PFOS	Electro- polymerization	Pulse Voltammetry	Molecularly imprinted polymer	20 ppt	50 ppt - 750 ppb	Low	Good	Simple	59
PFOA/PFOS	Bubble-Nucleation-Based Method	Voltammetry	Pt nanoelectrode	PFOS:.30 ppb<br>PFOA: 80 ppb	0.1–100 ppm	Low	Moderate	Moderate	19
PFOS	Impedimetric	Interdigitated microelectrode	Metal-organic framework	0.5 ppt	0.5ppt – 1 ppb	Low	Good	Moderate	18
PFOS	luminescent	spectrophotometer	Metal-organic framework	19 ppb	0-80 ppm	Low	Good	Moderate	60
PFOS	Colorimetric	Paper-based analytical	Methylene Green	10 ppm	1-500 ppm	Low	High	Simple	61
PFOA/PFOS	Colorimetry	Smartphone app	Methylene Blue	0.5 ppb	10-1000 ppb	Low	High	Simple	62
PFOS	Triple channel optical	Fluorescence, UV–vis absorption, and resonance light scattering	Carbon quantum dots	9.13 ppb	0–6 ppm	Low	Low	Moderate	63
PFOS	Colorimetric	Absorbance	MoS_2/Fe_3O_4 nanocomposites	4.3 ppb	0.05-6.25 ppm	Low	Moderate	Simple	16
PFOA/PFOS	Ionization and Mass measurement	HPLC-MS	Direct measurement	0.4 ppb	0-4 ppm	High	Low	Moderate	Control Group
PFOA/PFOS	Host-Guest interaction	Single molecule Nanopore	Hp-γ-CD	0.4 ppb	0-4 ppm	Low	High	Simple	This Work

Table S3. Calculated association constants for different CDs with different PFAS molecules. The method by Ramos Cabrer *et al.*[90] and nonlinear least-squares regression analysis were used for association constant determination* based on the chemical shift from ^{19}F NMR spectra.[67,70]

PFAS	Chemical Formula	Full Name	Abbreviations	Association Constants (M^{-1})			
				HP-γ-CD	γ-CD	β-CD	α-CD
PFCAs family	$C_4HF_7O_2$	Heptafluorobutyric acid	PFBA	107.07	-	-	-
	$C_5HF_9O_2$	Perfluoropentanoic acid	PFPA	132.03	-	-	-
	$C_6HF_{11}O_2$	Perfluorohexanoic acid	PFHxA	79.48	-	-	-
	$C_7HF_{13}O_2$	Perfluoroheptanoic acid	PFHpA	3758.26	-	-	-
	$C_8HF_{15}O_2$	Perfluorooctanoic acid	PFOA	698.90	832.66	10417	77.05
	$C_9HF_{17}O_2$	Perfluorononanoic acid	PFNA	1769.14	-	-	-
	$C_{10}HF_{19}O_2$	Perfluorodecanoic acid	PFDA	2119.58	-	-	-
PFSAs family	$C_4HF_9O_3S$	Perfluorobutanesulfonic acid	PFHxS	100.28	-	-	-
	$C_6HF_{13}O_3S$	Perfluorohexanesulphonic acid	PFBS	200.06	-	-	-
	$C_8HF_{17}O_3S$	Perfluorooctanesulfonate	PFOS	429.63	1017.55	12563	84.34

*Nonlinear least-squares regression analysis was implemented with MATLAB to estimate the association constants (K) according to the following equation:

$$y = \frac{A}{2a}\{a + x + \frac{1}{K} - \left(\left(a + x + \frac{1}{K}\right)^2 - 4ax\right)^{\frac{1}{2}}\}$$

y is the observed chemical shift less the free PFAS chemical shift; *a* is the concentration of all fluorinated species; *x* is the concentration of all CD species.

Table S4. The mean relative blockade ($\Delta I/I_0$) of CD and CD-PFAS (All values are fitted with a Gaussian equation. The margin of each value represents the standard deviation of $\Delta I/I_0$ from the Gaussian fitting).

	$\Delta I/I_0$ 1	$\Delta I/I_0$ 2	$\Delta I/I_0$ 3
α-CD	0.3184±0.1799	0.8901±0.0760	-
α-CD-PFOA	0.1575±0.1827	0.9029±0.0266	-
α-CD-PFOS	0.1443±0.1471	0.9023±0.0247	-
β-CD	0.7225±0.0421	-	-
β-CD-PFOA	0.0901±0.1141	0.4994±0.1824	-
β-CD-PFOS	0.1136±0.0753	-	-
β-CD-PFOA-PFOS	0.0763±0.0752	0.4667±0.3233	0.6975±0.0911
PFOA	0.08955 ± 0.0241	-	-
PFOS	0.1164 ± 0.0787	-	-

Table S5. Height (h), external (d_{ext}) and internal (d_{int}) diameters, predicted current (I), and predicted (f) and experimental (f^{exp}) blockade ratios for open α-HL and α-HL with different molecule systems in this study.

	h / nm	d_{ext} / nm	d_{int} / nm	I / pA	f	f^{exp}
α-HL				305		
α-HL + γ-CD	0.8	1.69	0.77	107	0.65	0.67
α-HL + β-CD	0.8	1.53	0.61	48	0.84	0.72
α-HL + α-CD	0.8	1.32	0.45	3	0.99	0.99
α-HL + HP-γ-CD	0.8	1.69	0.62	52	0.83	0.83
α-HL + PFOA						
off-axis	1.03	0.5		258	0.15	0.09
on-axis	1.03	0.5		200	0.34	
α-HL + PFOS						
off-axis	1.3	0.5		259	0.15	0.12
on-axis	1.3	0.5		200	0.34	
